# Gene Network Polymorphism Illuminates Loss and Retention of Novel RNAi Silencing Components in the *Cryptococcus* Pathogenic Species Complex

**DOI:** 10.1371/journal.pgen.1005868

**Published:** 2016-03-04

**Authors:** Marianna Feretzaki, R. Blake Billmyre, Shelly Applen Clancey, Xuying Wang, Joseph Heitman

**Affiliations:** Department of Molecular Genetics and Microbiology, Duke University Medical Center, Durham, North Carolina, United States of America; University of California-Riverside, UNITED STATES

## Abstract

RNAi is a ubiquitous pathway that serves central functions throughout eukaryotes, including maintenance of genome stability and repression of transposon expression and movement. However, a number of organisms have lost their RNAi pathways, including the model yeast *Saccharomyces cerevisiae*, the maize pathogen *Ustilago maydis*, the human pathogen *Cryptococcus deuterogattii*, and some human parasite pathogens, suggesting there may be adaptive benefits associated with both retention and loss of RNAi. By comparing the RNAi-deficient genome of the Pacific Northwest Outbreak *C*. *deuterogattii* strain R265 with the RNAi-proficient genomes of the *Cryptococcus* pathogenic species complex, we identified a set of conserved genes that were lost in R265 and all other *C*. *deuterogattii* isolates examined. Genetic and molecular analyses reveal several of these lost genes play roles in RNAi pathways. Four novel components were examined further. Znf3 (a zinc finger protein) and Qip1 (a homolog of *N*. *crassa* Qip) were found to be essential for RNAi, while Cpr2 (a constitutive pheromone receptor) and Fzc28 (a transcription factor) are involved in sex-induced but not mitosis-induced silencing. Our results demonstrate that the mitotic and sex-induced RNAi pathways rely on the same core components, but sex-induced silencing may be a more specific, highly induced variant that involves additional specialized or regulatory components. Our studies further illustrate how gene network polymorphisms involving known components of key cellular pathways can inform identification of novel elements and suggest that RNAi loss may have been a core event in the speciation of *C*. *deuterogattii* and possibly contributed to its pathogenic trajectory.

## Author Summary

Genome instability and mutations provoked by transposon movement are counteracted by novel defense mechanisms in organisms as diverse as fungi, plants, and mammals. In the human fungal pathogen *Cryptococcus neoformans*, an RNAi silencing pathway operates to defend the genome against mobile elements and transgene repeats. RNAi silencing pathways are conserved in the *Cryptococcus* pathogenic species complex and are mediated by canonical RNAi components. Surprisingly, several of these components are missing from all analyzed *C*. *deuterogattii* VGII strains, the molecular type responsible for the North American Pacific Northwest outbreak. To identify novel components of the RNAi pathways, we surveyed the reference genomes of *C*. *deuterogattii*, *C*. *gattii*, *C*. *neoformans*, and *C*. *deneoformans*. We identified 14 otherwise conserved genes missing in R265, including the *RDP1*, *AGO1*, and *DCR1* canonical RNAi components, and focused on four potentially novel RNAi components: *ZNF3*, *QIP1*, *CPR2*, and *FZC28*. We found that Znf3 and Qip1 are both required for mitotic- and sex-induced silencing, while Cpr2 and Fzc28 contribute to sex-induced but not mitosis-induced silencing. Our studies reveal elements of RNAi pathways that operate to defend the genome during sexual development and vegetative growth and illustrate the power of network polymorphisms to illuminate novel components of biological pathways.

## Introduction

Genome reduction is a common adaptation among bacterial pathogens and commensals, and has been hypothesized to occur for a number of reasons, including increased specificity to a host or environmental range, or to increase virulence more directly through loss of an antivirulence gene or gene cluster. The former case can be explained primarily through loss of genes that play only accessory roles. These genes can become dispensable as an organism becomes obligately associated with a host, which then acts as an alternative source for these gene products, such as amino acids or metabolic intermediates [1–3]. In some cases, network polymorphisms can result from loss of one of the components, which then enables additional inactivating mutations to occur in other components of the crippled or disabled pathway, such as loss of the Gal80 repressor in *Saccharomyces kudriavzevii* [4]. Genes also can be lost as a result of an “antivirulence” function, as is seen in *Shigella* and *E*. *coli*, where the presence of the lysine decarboxylase *cadA* interferes with the synthesis of enterotoxins through production of cadaverine [5]. This model, termed the black hole hypothesis, suggests that gene losses can be the result of active interference with pathogenesis, likely as the result of gain of a new incompatible function. In either model, understanding the gene network polymorphism can elucidate the biology and evolution of the pathogen, facets that are particularly relevant for new and emerging pathogens.

*Cryptococcus deuterogattii*, previously *C*. *gattii* molecular type VGII [6], is an emerging human fungal pathogen in the Pacific Northwest (PNW) of the United States and southwest Canada [7–9]. While the sibling species *C*. *neoformans* predominantly infects immunocompromised individuals, many of the *C*. *deuterogattii* infected patients in the Pacific Northwest outbreak were otherwise healthy. Both species cause severe pulmonary and central nervous system infections, and are fatal if untreated. Surprisingly, whole genome sequencing revealed that the *C*. *deuterogattii* strain R265 is missing both of the Argonaute genes, essential components of the RNAi-induced silencing complex (RISC) [10,11]. Further examination revealed that in addition to the loss of both Argonaute genes, one of the two Dicers and the only RNA-dependent RNA polymerase have also undergone pseudogenization through large sequence losses similar to those of the Argonaute genes [12]. The loss of critical canonical components of the RNAi pathway raises a number of questions about the origins and biology of the *C*. *deuterogattii* species as well as the function of RNAi within the *Cryptococcus* pathogenic species complex as a whole.

RNA interference (RNAi) is a highly conserved mechanism among eukaryotes that facilitates homology-dependent gene silencing. This transcriptional regulatory strategy was initially observed in *Caenorhabditis elegans* where exogenously introduced double-stranded RNA (dsRNA) triggers silencing of the transcript complementary to the dsRNA sequence [13]. Since its discovery in *C*. *elegans*, numerous species of plants, animals, fungi, and protists have been found to employ similar strategies to either protect their genomes from foreign DNA or to orchestrate gene expression and diverse cellular, developmental, and physiological processes [14–17]. Repetitive sequences are often found in mobile genetic elements and previous studies found an association between RNAi and transposable elements, which are ubiquitous in eukaryotic organisms. Transposon activation and movement impairs genome stability and increases the mutational burden of the host. Therefore, eukaryotes employ different strategies to inhibit and limit transposon expansion. *Arabidopsis thaliana*, *Drosophila melanogaster*, *Saccharomyces castellii*, *Neurospora crassa*, and *C*. *elegans* all utilize RNAi strategies to control and inhibit transposon expression [18–22].

*C*. *neoformans* also employs an RNAi-related pathway to inhibit transposable elements. In previous studies, Wang *et al*. showed that the insertion of a tandem multicopy transgene triggered a homology-dependent gene silencing mechanism during sexual development and termed this process sex-induced silencing (SIS) [10]. This process was identified specifically with a *SXI2***a**-*URA5* transgene array inserted into the *ura5* locus, resulting in the presence of three functional copies of *URA5* and one nonfunctional copy. During mating, progeny that inherit the array silence the *URA5* gene in an RNAi-dependent manner approximately 50% of the time. In addition, Wang *et al*. later found that transgene silencing can also occur during vegetative growth, named mitotic-induced silencing (MIS), but at a relatively lower frequency in mitotic (~0.2%) compared to meiotic progeny (~50%) [23]. Further analysis showed that SIS and MIS require the RNAi components Rdp1 (RNA-dependent RNA polymerase), Ago1 (Argonaute), and Dcr1/2 (dicer-like proteins) [10,23]. SIS and MIS function to inhibit transposon movement and thus serve as a genome defense mechanism during meiosis and mitosis. The initial observation of transposon silencing during sexual development was made in the highly virulent *C*. *neoformans* lineage. Later studies found that transgene-related SIS also occurs in *C*. *deneoformans* and that the RNAi components are required for transposon silencing during both bisexual and unisexual development [24].

The lack of the critical Argonaute, Dicer, and RdRp components of the RNAi pathway in *C*. *deuterogattii* suggests that the loss of RNAi may represent a gene network polymorphism. In fact, the RNAi pathway is intermittently conserved and lost across eukaryotes [12,25–27]. In *Leishmania* and trypanosomes, RNAi losses were previously taken advantage of in order to identify additional, previously unknown components of the RNAi pathway via comparative genomics [16]. To test the hypothesis that the RNAi pathway represents a gene network polymorphism, we surveyed the genomes of the R265 (*C*. *deuterogattii*), WM276 (*C*. *gattii*), H99 (*C*. *neoformans*), and JEC21 and B-3501A (*C*. *deneoformans*) strains and found 14 genes missing from *C*. *deuterogattii*, including the canonical components of the RNAi pathway *RDP1*, *AGO1*, and *DCR1*. Here we focus on four of these lost components: *ZNF3*, previously identified as a regulator of hyphal development during unisexual and bisexual reproduction [28]; *CPR2*, a G-protein coupled receptor (GPCR) previously studied for its role as an accessory constitutively active pheromone receptor [29]; *QIP1*, independently identified as an RNAi component via a mass spectrometry approach [30]; and *FZC28*, a putative transcription factor with no obvious phenotypes in a systematic genome-wide transcription factor deletion study [31].

Here we demonstrate that the loss of the RNAi components represents a bona fide system polymorphism, with several previously unknown RNAi components lost in *C*. *deuterogattii*. In addition, we show that mutants of these missing genes in *C*. *neoformans* fall into two classes: mutants that lose both vegetative silencing and sex-induced silencing, and mutants that are affected only in the frequency of sex-induced silencing. This suggests that sex-induced silencing may be a specialized, highly induced variant of the vegetative transgene-induced silencing pathway, rather than a separate pathway. Taken together, our results show that a substantial loss of genes contributing to two related RNAi pathways has occurred in *C*. *deuterogattii*. By using comparative genomics, these gene losses reveal key insights that aid in elucidating the functions of these RNAi-based genome conservation pathways.

## Results

### RNAi components are missing from *C*. *deuterogattii*

**Fig 1:**
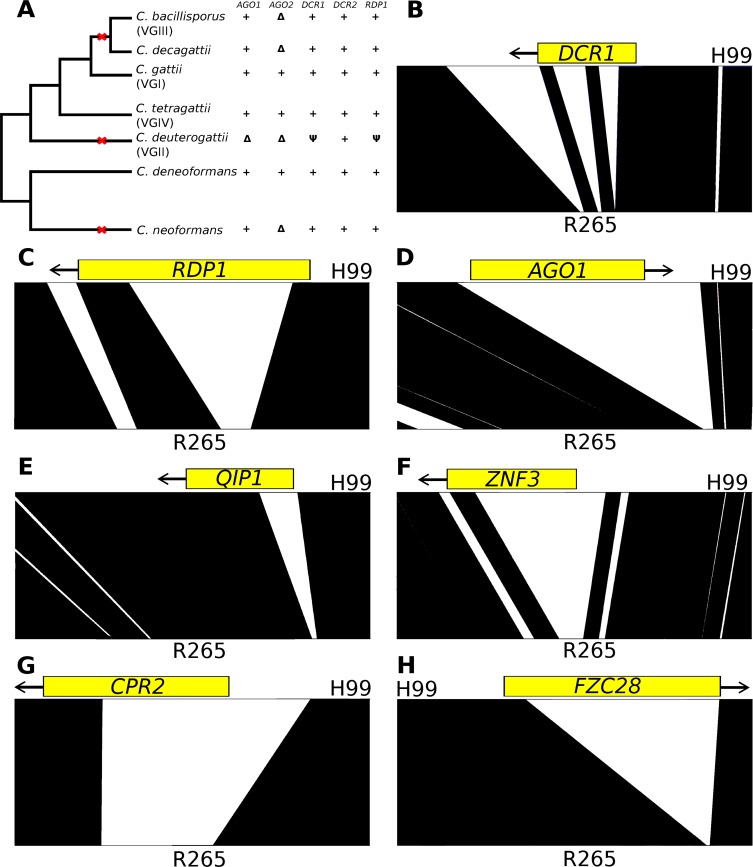
Genes conserved in the pathogenic species complex but lost in the VGII *C*. *deuterogattii* lineage. A) The known canonical components of RNAi have been broadly conserved in the pathogenic species complex, with the exception of the *AGO2* gene. Red X’s mark independent losses of RNAi components. In contrast, the *C*. *deuterogattii* lineage has lost 4 of the 5 canonical components. Delta symbols indicate a complete deletion while a psi indicates a pseudogenization event. B-H) Gene losses in canonical components and in genes shown here to influence the RNAi pathway are visualized using ACT. Blastn alignments were utilized to align the H99 and R265 genomes. Large portions of these genes have been cleanly lost, in some cases comprising the entire ORF (D) and in others including the start codon (E,F,G).

The *C*. *deuterogattii* lineage (previously VGII *C*. *gattii*) is responsible for the recent, ongoing outbreak on Vancouver Island and its expansion into the Pacific Northwest of the United States. Initial analysis of the R265 *C*. *deuterogattii* reference genome revealed that both the key canonical RNAi components *AGO1* and *AGO2* are missing, indicating that the VGII lineage of *C*. *deuterogattii* may lack a functional RNAi pathway [10,11]. Upon further examination, we discovered that two of the other canonical components, *DCR1* and *RDP1*, had both suffered truncations removing key functional domains and are therefore pseudogenes. Of the known RNAi canonical components, only *DCR2* remains intact in *C*. *deuterogattii* (Fig 1A) [11,32–35]. We hypothesized that this loss of multiple RNAi components may represent a gene network polymorphism where all of the components of a pathway are intact in one species, but have been selectively lost in another closely related species. We further hypothesized that a whole genome comparison of *C*. *deuterogattii* with other related *Cryptococcus* species would reveal novel components of the RNAi pathway lost in *C*. *deuterogattii* but otherwise maintained throughout the pathogenic species complex.

We compared the publicly available reference genomes of JEC21 (*C*. *deneoformans*)[36], B-3501A (*C*. *deneoformans*)[36], H99 (*C*. *neoformans*)[37], and WM276 (*C*. *gattii*)[11] with R265 (*C*. *deuterogattii*)[11] to identify otherwise conserved genes that were missing or truncated in the *C*. *deuterogattii* lineage. We found seven conserved genes that were not annotated in R265 and seven others that were dramatically shortened (over 50% different in length) as a result of extensive deletions of genomic sequence (Table 1). All 14 genes were lost across the entire VGII group, based on 53 publicly available whole genome sequences from *C*. *deuterogattii* [32]. These genome sequences did reveal some diversity in these regions. Estimation of Tajima’s D in windows across the genome and within the regions left by the deletion events showed a highly negative value for the genome as a whole (mean of -1.122), and a slightly more positive (mean of -0.796), but not statistically significant value (p = 0.0901) for the deletion windows (S1 Fig). We did not identify any transposable elements or repeats that may have mediated the deletion events. One of the seven missing genes was the previously identified canonical RNAi component *AGO1*. In each case, localized deletions of sequence occurred, encompassing entire ORFs, start codons, and/or functional domains of the candidate genes (Fig 1B–1H and S2 Fig). Our screen identified two potential transcription factors, *FZC27* and *FZC48*, and three genes, including *GWC1*, *GWO1*, and *QIP1*, which have been previously identified as participating in the degradation of unspliced mRNA through RNAi [30]. Two of the 14 missing or truncated genes, *CPR2* and *ZNF3*, were previously shown to play roles in unisexual and bisexual reproduction, but were not described as having a role in RNAi [17,35]. We chose to focus on four genes as candidates to interrogate for a role in the SIS and MIS RNAi pathways: *CPR2*, *FZC28*, *ZNF3*, and *QIP1*.

**Table 1:** List of genes identified in comparative genomic screen.

Name	Accession Number	Description	Annotated in R265	Role in RNAi
*CPR2*	CNAG_03938	constitutively active receptor related to Ste3 pheromone receptor	Yes	SIS
*DCR1*	CNAG_02742	canonical Dicer	Yes	SIS/MIS
*QIP1*	CNAG_01423	homolog of QIP	Yes	SIS/MIS
*RDP1*	CNAG_03466	canonical RdRp	Yes	SIS/MIS
*FZC47*	CNAG_04184	fungal transcription factor	Yes	none
*GWC1*	CNAG_06486	GW/WG protein	Yes	Dumesic et al. [30]
*MEH1*	CNAG_06497	microsomal epoxide hydrolase	Yes	unknown
*FZC28*	CNAG_00505	fungal transcription factor	No	SIS
*GWO1*	CNAG_00520	GW182-like	No	Dumesic et al. [30]
*CDP1*	CNAG_03734	chromodomain protein	No	unknown
*ZNF3*	CNAG_02700	zinc finger protein	No	SIS/MIS
*OXR1*	CNAG_06609	oxidoreductase	No	unknown
*FCR1*	CNAG_06976	ferric-chelate reductase	No	none
*AGO1*	CNAG_04609	canonical Argonaute	No	SIS/MIS

Genes that were identified as lost or substantially changed in length in the R265 genome, along with their H99 accession numbers. Genes were assayed for a role in RNAi, as described below.

*CPR2* encodes a seven transmembrane domain GPCR closely related phylogenetically to the Ste3 family of pheromone receptors, but it is constitutively active and independent of pheromone ligand binding [29]. Cpr2 signals via the same G proteins as the pheromone receptor Ste3, and overexpression of *CPR2* can rescue the sterility defect of *ste3*Δ mutants, although it may bias cells towards unisexual reproduction [29]. *FZC28* is a transcription factor about which very little is known. It was identified and mutated as part of a genome-wide transcription factor deletion library, and experiments in that study identified no obvious phenotypes [31].

In previous studies we found that *ZNF3* is required for hyphal development during unisexual and bisexual reproduction in *C*. *deneoformans* [28]. Deletion of the gene blocks hyphal development and impairs pheromone expression during mating. However, it does not play a direct role in the pheromone-signaling cascade. Surprisingly, microarray expression analysis revealed that deletion of Znf3 increased transposon and transposon-related gene expression during bisexual reproduction [28]. Znf3 is also somewhat rapidly diverging in amino acid sequence. While it is found in the *Cryptococcus* pathogenic species complex and the neighboring *sensu stricto* (including *C*. *amylolentus*) and *sensu lato* groups (including *C*. *heveanensis*), the sequence is not well conserved, and it shares only weak homology over a 211 amino acid stretch (23% identity and 38% positive) with the reciprocal best BLAST hit ortholog in *Tremella mesenterica*. The encoded protein in *Cryptococcus neoformans* contains three zinc finger domains, two predicted nuclear localization signals (NLS), and a conserved coiled coil region, often involved in protein-protein interactions, as well as a putative ribonuclease conserved domain indicating that it may be involved in cleavage of RNA.

*QIP1* is named for *N*. *crassa QIP*, which functions during quelling and MSUD by binding to RISC and stimulating cleavage of the passenger strand of the duplex siRNA [38]. Moreover, a previous study directly implicated Qip1 in the transcriptional squelching of transposons and the degradation of mRNAs that have poorly spliced non-canonical introns [30]. Dumesic *et al*. localized Qip1 in the nucleus and showed that it physically interacts with Rdp1 as part of the Spliceosome-Coupled and Nuclear RNAi (SCANR) complex [30]. Analysis of *N*. *crassa* Qip revealed a conserved 3’-5’ exonuclease domain belonging to the DEDDh superfamily of nucleases, showing high similarity to the *E*. *coli* DNA polymerase III ε subunit [33]. Although, the *C*. *neoformans* Qip1 protein does not contain any detected conserved functional domains, it exhibits weak similarity to the helical domain of Class IIa histone deacetylases, which may suggest a role different than that of *N*. *crassa* Qip.

### Non-canonical components of RNAi pathway

In previous studies, Wang *et al*. found that a tandem multicopy insertion of a *SXI2***a**-*URA5* transgene triggered silencing of the *URA5* gene during bisexual reproduction and vegetative growth in *C*. *neoformans* [10,23]. When F1 progeny were isolated from a cross between WT *MAT*α *URA5* (H99α) and *MAT***a** *SXI2***a**-*URA5* (JF289), ~25% were found to be uracil-auxotrophic despite the fact that all of them had intact copies of the *URA5* allele. Further analysis revealed that ~50% of the progeny that inherited the *SXI2***a**-*URA5* transgene were uracil auxotrophic. Recent studies showed that the transgene induced silencing mechanism is activated efficiently during bisexual and unisexual reproduction (SIS) and less efficiently during vegetative growth (MIS) [23,24].

Deletion of RNA-dependent RNA-polymerase Rdp1 abolished transgene induced silencing during SIS and MIS in both *C*. *neoformans* and *C*. *deneoformans*. To investigate the role of the missing genes from R265 in silencing we generated deletion mutants in the JF289**a** isolate bearing the *SXI2***a**-*URA5* transgene (derived from strain KN99**a**), and the congenic WT H99α strain. Two independent deletion mutants for each gene were isolated and analyzed.

**Fig 2:**
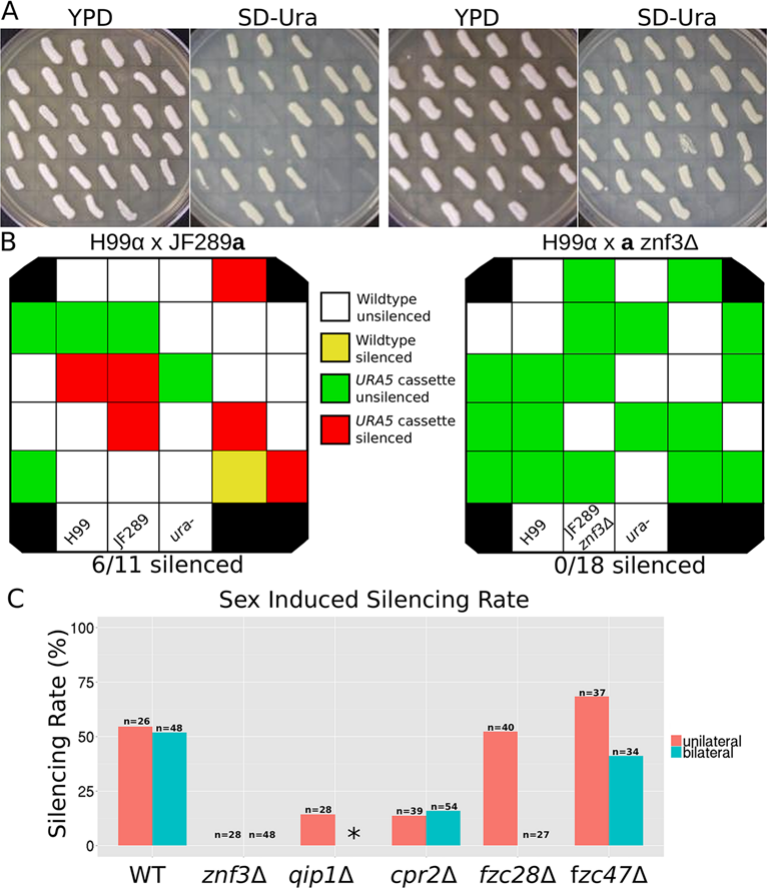
Non-canonical components are required for sex-induced silencing via RNAi. (A) Deletion of *ZNF3* and *QIP1* significantly reduces silencing in unilateral mutant matings. Progeny from wild type and unilateral mutant matings (one parent is mutant) were isolated and evaluated for *URA5* silencing by growth on rich media (YPD) and SD-uracil. The parents and *ura5* mutant were included as controls. The plates were incubated at 30°C for 3 days. Strains used: H99α crossed with JF289**a** and H99α crossed with XW205. (B) Schematic indicating the presence or absence of the transgene array in the spores from (A) as tested by PCR (Primary data in S3 Fig), as well as the occurrence of silencing. (C) Frequency of sex-induced silencing in all mutants tested. Primary data is shown in S4 Fig. The asterisk indicates that only unilateral silencing was tested for *qip1*Δ mutants because bilateral matings did not produce viable spores. Strains used: for *znf3*Δ: MF65 and XW205; for *qip1*Δ: SEC1, SEC2, SEC3, SEC4, and H99; for *cpr2*Δ: YPH16, XW197, JF289**a**; for *fzc28*Δ: SEC7, SEC8, YSB2337, YSB2338, H99; and for *fzc47*Δ: SEC5, SEC6, YSB1406, YSB1407, and H99.

To determine the silencing efficiency of the mutants during sexual reproduction, unilateral (one parent is mutant) and bilateral (both parents are mutants) crosses were performed on MS media. We dissected random F1 spore progeny from each cross and these were tested for growth in the absence of uracil and genotyped for the presence of the *SXI2***a**-*URA5* transgene (Fig 2A and S3 Fig). In unilateral matings with a deletion allele only present in one of the two parents, two meiotic progeny were ura^-^ for *qip1*Δ (out of 14 inheriting the array, ~14%), none were ura^-^ for *znf3*Δ (out of 18 inheriting the array, 0%), and three were ura- for *cpr2*Δ (out of 22 inheriting the array, ~13.6%) indicating significantly reduced silencing efficiency compared to WT (Fig 2A and S4 Fig). These results suggest that all three components play a role in RNAi during sexual development. In contrast, the silencing efficiency of the *SXI2***a**-*URA5* transgene in the *fzc28*Δ, and *fzc47*Δ unilateral mutant matings was similar to WT (~50%) (Fig 2C). All of the ura^-^ progeny carry an intact copy of the *SXI2***a**-*URA5* transgene, as verified by PCR.

Previous studies showed that bilateral matings of all three canonical RNAi component mutants *(ago1*Δ, *dcr1*Δ, *rdp1*Δ) yielded ~20 fold fewer spores, with *rdp1*Δ mutants also demonstrating disorganized and atypical basidia, but with no effect on the sporulation efficiency of the spores that were produced [10]. Similarly, although deletion of *ZNF3* severely impaired mating in *C*. *deneoformans* [28], hyphal development during bisexual reproduction was similar to WT in *C*. *neoformans znf3*Δ mutants, albeit somewhat delayed. In contrast, in bilateral *qip1*Δ x *qip1*Δ mutant crosses we found that spore production was severely impaired and the few spores that were isolated failed to germinate, indicating that Qip1 is required for completion of the sexual cycle and may play a role in meiosis (S4B Fig). On the other hand, deletion of *RDP1* or *ZNF3* did not affect sporulation efficiency. Deletion of *ZNF3* in both parents completely abolished silencing, as none of the progeny that inherited the transgene were ura^-^ (S4A Fig). These results indicate that Znf3 is required for silencing during mating and deletion of the gene causes a severe SIS silencing defect, similar to *rdp1*Δ. Silencing of the *URA5* gene was also impaired in *fzc28*Δ and *cpr2*Δ bilateral matings (Fig 2C). However, *fzc47*Δ mutation in both parents did not impair silencing of the *URA5* transgene and it was similar to WT, despite a modest increase in silencing rate in a unilateral cross (Fig 2C and S4 Fig).

**Fig 3:**
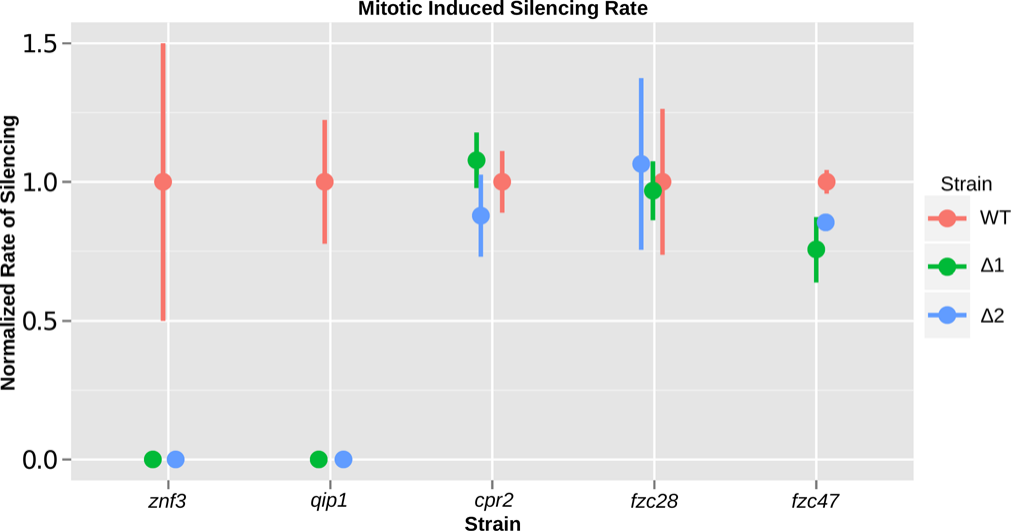
Non-canonical components are required for mitotic-induced silencing via RNAi. Znf3 and Qip1 are required for *URA5* repetitive transgene silencing during vegetative growth. Wild type JF289**a** isolate and the derived deletion mutants were grown in rich media and plated on 5-FOA to calculate the frequency of *URA5* silencing. The silencing frequency is normalized to the frequency of the wild type JF289 strain and plotted with error bars indicating the standard deviation from the mean. The other three mutants tested showed only minor non-significant variation in silencing rate from the wild type. Strains used: JF289**a** (wild type), XW205 and XW206 (*znf3*Δ), SEC1 and SEC2 (*qip1*Δ), XW197 and XW198 (*cpr2*Δ), SEC7 and SEC8 (*fzc28*Δ), and SEC5 and SEC6 (*fzc47*Δ).

We then examined the silencing frequency of the *SXI2***a**-*URA5* transgene in the mutant strains by measuring spontaneous 5-FOA resistance following mitotic growth in rich media. The strains bearing the *qip1*Δ and *znf3*Δ deletions failed to yield any colonies on 5-FOA media, indicating that these two genes are required for transgene-induced mitotic silencing (Fig 3). In contrast, deletion of two transcription factors, *FZC28* and *FZC47*, obtained from a recently reported systematic transcription factor deletion collection and crossed into the JF289 background [31], and the GPCR *CPR2*, did not alter the mitotic silencing frequency of the *SXI2***a**-*URA5* transgene compared to WT.

In conclusion, we found that Znf3 and Qip1 are required for silencing during both MIS and SIS and deletion of the genes generates a phenotype similar to mutation of *RDP1*, whose gene product is essential for RNAi function in *C*. *neoformans*. These results suggest that Znf3 and Qip1 are novel regulators or components of the RNAi pathway. In addition we found that a new transcription factor Fzc28 and the GPCR Cpr2 influence transgene-induced silencing specifically during sexual development, possibly coupling the sexual cycle with the RNAi pathway but likely not acting mechanistically during silencing itself.

### Znf3 and Qip1 are required for transposon suppression

**Fig 4:**
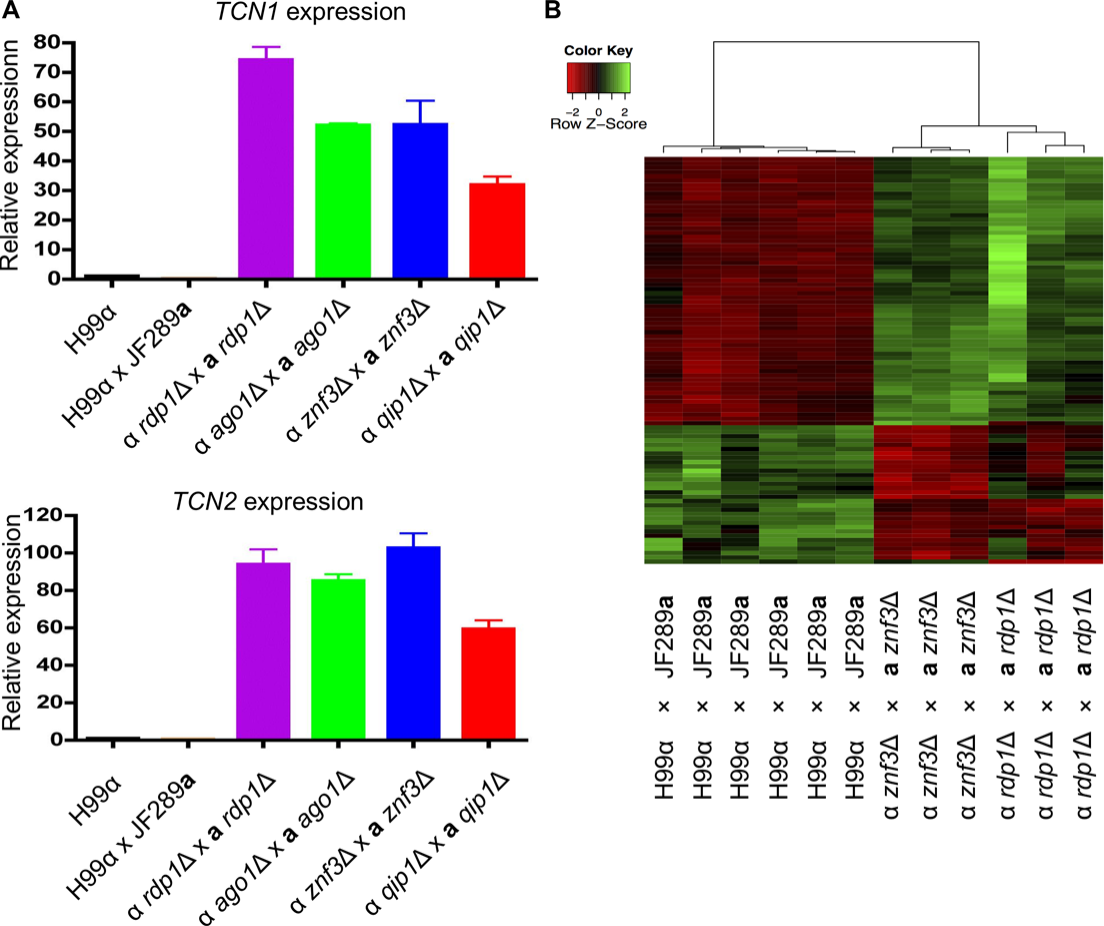
Znf3 is required for transposon silencing. (A) Quantitative real-time PCR showed that deletion of Znf3 or Qip1 resulted in markedly increased abundance of transposon-derived transcripts, similar to mutation of other canonical components of the RNAi pathway. Strains used: H99 and JF289**a** (wild type), YPH348 and YPH351 (*rdp1*Δ), YPH738 and YSB299 (*ago1*Δ), XW205 and MF65 (*znf3*Δ), and SEC1 and SEC3 (*qip1*Δ). (B) Heat map of differentially expressed genes in a wild type cross versus a bilateral *znf3*Δ x *znf3*Δ cross or a bilateral *rdp1*Δ x *rdp1*Δ cross. Strains used: H99 and JF289**a** (wild type), XW205 and MF65 (*znf3*Δ), YPH348 and YPH351 (*rdp1*Δ).

In a previous study we found that deletion of Znf3 in *C*. *deneoformans* activates transposon expression [28] and here we have shown that it is required for MIS and SIS. Recent studies revealed that transposable element expression increases during sexual reproduction and the components of the RNAi pathway maintain genome integrity through an efficient transposon silencing mechanism [10]. Deletion of *RDP1* results in centromeric and telomeric retrotransposon overexpression during sexual development in *C*. *neoformans* [10]. We examined the transcript abundance of two transposons, Tcn1 and Tcn2, in *znf3*Δ mutant crosses and found that abundance was dramatically increased, similar to *rdp1*Δ and *ago1*Δ mutant crosses (Fig 4A). Deletion of *QIP1* also yielded elevated levels of transposon transcript abundance, indicating that Qip1 also plays a major role in transposon quenching during sexual development (Fig 4A).

To further investigate the role of Znf3 in transposon silencing on a genome-wide scale, we performed a comparative transcriptome analysis of *znf3*Δ x *znf3*Δ and *rdp1*Δ x *rdp1*Δ crosses during sexual development and vegetative growth. Bilateral crosses of *znf3*Δ x *znf3*Δ and *rdp1*Δ x *rdp1*Δ mutants were incubated on solid V8 medium (pH = 5) for 24 hours, as well as H99α x JF289**a** wild type crosses. RNA was isolated from the mating cultures, transcribed to cDNA, and hybridized to a *C*. *neoformans* genome microarray.

Genome-wide expression analysis revealed that among the transcripts with altered expression level, the majority were increased in the *znf3*Δ mutant cross relative to WT during sexual development, indicating that Znf3 has a repressive role during sexual development. The few transcripts whose abundance was decreased in the *znf3*Δ and *rdp1*Δ crosses are involved in hypoxia, oxidation, ion channels, sugar transport, and possibly sporulation. During *znf3*Δ sexual development more than 80 independent microarray tags exhibited a twofold increase in abundance compared with the WT. Further analysis revealed that the majority of these tags correspond to sequences from hypothetical proteins or align to intergenic regions of the *C*. *neoformans* H99 genome. Alignment to a retrotransposon library [39] showed that almost all of the intergenic probes that were increased in *znf3*Δ mutants correspond to retrotransposon sequences found in multiple sites in the genome (S3 Table). We found that these retrotransposons have long terminal repeats (LTR) and reside in the centromeric and telomeric regions of the chromosomes. In addition, most of the upregulated hypothetical proteins in *znf3*Δ x *znf3*Δ crosses were found to be RNA and DNA helicases, RNA-dependent DNA polymerases, and other transposon-related proteins (S3 Table). During vegetative growth fewer transcripts were upregulated in *znf3*Δ mutants; however, the transcripts that exhibited differential abundance were also involved in transposon expression or activation. As was observed previously, the Tcn1, Tcn2, and Tcn3 elements were increased in *znf3*Δ × *znf3*Δ crosses, while their abundance was diminished during *znf3*Δ vegetative growth but remained significantly higher than the WT. We compared the transcriptional profile to the *rdp1*Δ x *rdp1*Δ mutant cross profile, and the whole genome transcript profiles between the two mutants were highly similar (Fig 4B). The highly correlated transcript profiles of upregulated genes suggests that Znf3 and Rdp1 have similar functions and may mediate retrotransposon silencing through the same RNAi pathway.

Interestingly, in spite of the loss of RNAi components in *C*. *deuterogattii*, transposon copy number does not appear to have dramatically increased in the genome (S5 Fig). The vast majority of transposable elements are present at substantially lower copy number in *C*. *deuterogattii* compared to *C*. *gattii*. However, several classes of transposable elements are present in approximately equal amounts (TCN3 and TCN6) or at even higher levels (TCN4 and LTR13) in *C*. *deuterogattii* (R265) than in *C*. *gattii* (WM276), based on a BLAST search using a *C*. *neoformans* library [39].

### Znf3 and Qip1 are sexually induced

**Fig 5:**
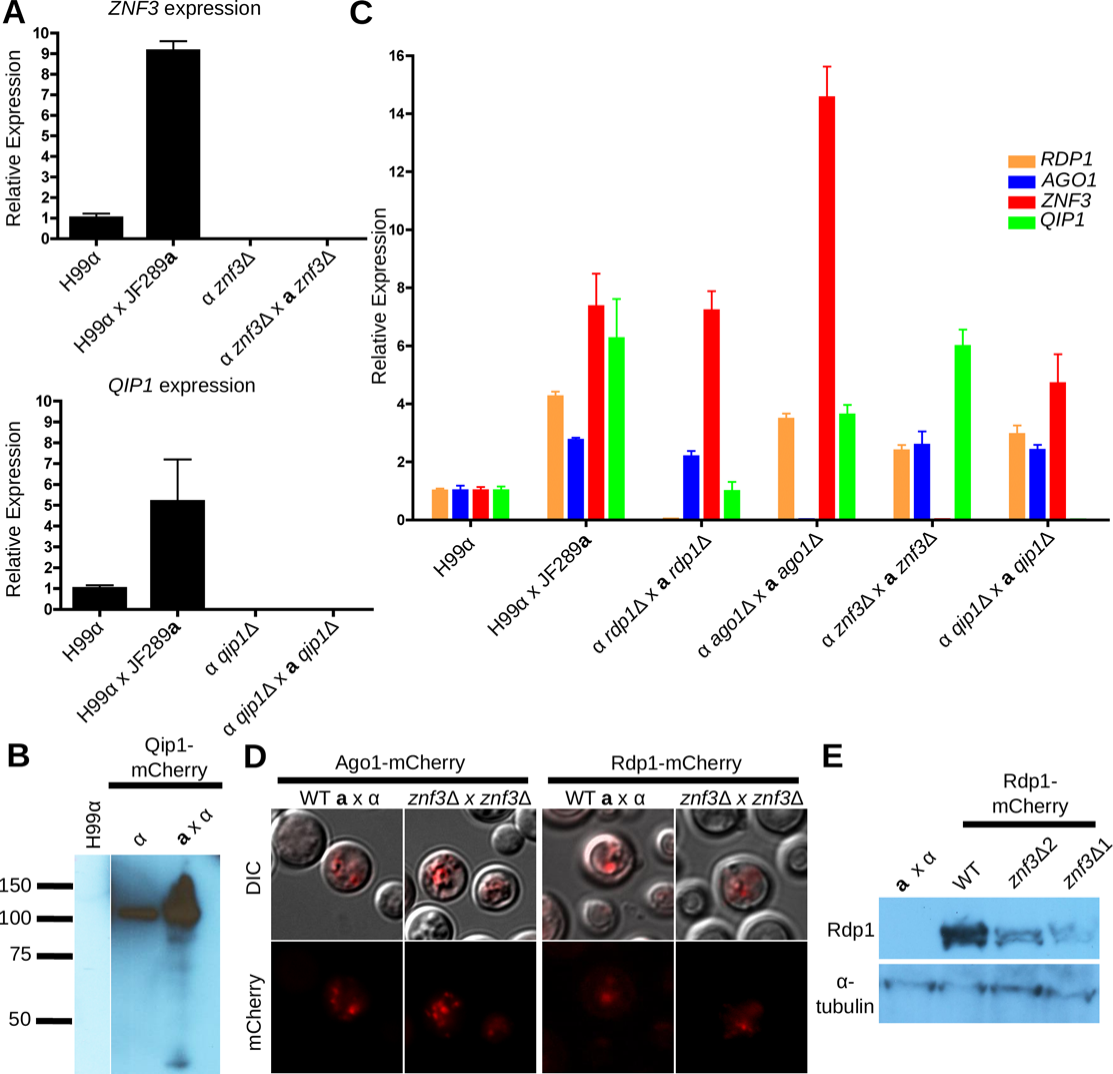
*ZNF3* and *QIP1* expression increases during mating. (A) Expression of *ZNF3* and *QIP1* was determined using qPCR during vegetative growth and during mating. Expression of both increased in mating conditions. Strains used for *ZNF3* expression: H99, H99 x JF289**a**, MF65, and XW205 x MF65. Strains used for *QIP1* expression: H99, H99 x JF289**a**, SEC3, and SEC1 x SEC3. B) Increased protein levels of Qip1 were also detected via western blot analysis during mating. Strains used: H99, MF190, and MF190 x JF289**a**. (C) Expression of the canonical RNAi components and *ZNF3* were determined via qPCR in bilateral crosses with mutations of the RNAi components. Loss of *ago1*Δ resulted in increased expression of *ZNF3*. Strains used: H99, H99 x JF289**a**, YPH348 x YPH351 (*rdp1*Δ), YPH738 x YSB299 (*ago1*Δ), MF62 x MF65 (*znf3*Δ), and SEC1 x SEC3 (*qip1*Δ). (D) Ago1-mCherry and Rdp1-mCherry direct fluorescence signals in wild type vs. *znf3*Δ x *znf3*Δ co-cultures under mating conditions. Mating mixtures were spotted and incubated on V8 (pH = 5) plates for 24 hours in the dark and then cells were scraped off and resuspended in water for microscopy. Strains used for Ago1: XW35 x YL99**a** and MF201 x MF62. Strains used for Rdp1-mCherry: XW37 x YL99**a** and MF197 x MF62. (E) Western blotting for Rdp1-mCherry shows that levels of Rdp1 are modestly decreased in a *znf3*Δ mutant compared to wild type. Strains used: H99 x JF289**a**, XW37 x JF289**a**, MF197 x MF62, and MF198 x MF62.

In previous studies we found that, although Znf3 regulates sexual development, *ZNF3* expression remains stable during vegetative growth and mating in *C*. *deneoformans* [28]. In addition, mRNA levels for the RNAi components are relatively similar between mitotic growth and mating based on northern blot analysis; however, their protein abundance was significantly higher during sexual development suggesting that the RNAi components are translationally induced or stabilized during the sexual cycle [10]. Based on this evidence we hypothesized that *ZNF3* and *QIP1* expression might also remain the same between the two conditions in *C*. *neoformans*. RNA was isolated during mitotic growth and mating from WT and bilateral mutant crosses and the abundance of their transcripts was analyzed using quantitative RT-PCR. Unlike the canonical RNAi components, we found that both *ZNF3* and *QIP1* expression was significantly higher during mating compared to WT (Fig 5A). This was a surprising result given that the expression of the highly conserved *ZNF3* gene in *C*. *deneoformans* remains the same and similar to WT during both conditions [28]. Moreover, Znf3 and Qip1 have similar roles with the RNAi components in SIS and MIS whose expression remains stable. This indicates that Znf3 and Qip1 expression may have a unique mode of regulation distinct from Rdp1 and Ago1.

We next assessed whether the RNA abundance during sexual development is correlated with the protein level between the two conditions. The C-termini of Znf3 and Qip1 were fused with mCherry at the endogenous genomic loci. MIS and SIS assays were conducted to test if the chimeric proteins retain their functional roles in silencing. Znf3 tagged with mCherry was completely defective in SIS and MIS, indicating that the mCherry tag interferes with function. On the other hand, Qip1 tagged with mCherry exhibited wild type levels of silencing during vegetative growth and sexual development. The protein levels were examined during both conditions and we found that, although the Qip1 protein was present during both vegetative growth and mating, it was significantly more abundant during sexual development, similar to the difference observed in RNA abundance (Fig 5B). These results indicate that Qip1, and possibly also Znf3, have a unique mode of regulation that is possibly distinct from that of other RNAi components.

### RNAi component regulation is independent of Qip1 and Znf3

The MIS and SIS silencing phenotypes of *znf3*Δ and *qip1*Δ mutants are very similar to *rdp1*Δ mutants. Previous studies have suggested that an unknown RNA-binding factor may govern translational regulation of the transcripts of the RNAi components to result in elevated protein levels specifically during sexual development [10]. We found that Znf3 bears both zinc fingers and an RNase domain and transcription of the gene is sexually induced. Considering that Znf3 has a similar phenotype to Rdp1, it could be involved in the translational regulation of the RNAi components, and the severe loss of silencing phenotype of *znf3*Δ mutants might be attributable to an absence of these factors.

It is unlikely that Znf3 regulates the transcription of the RNAi components based on microarray expression analysis. Nevertheless, we performed quantitative RT-PCR in the absence of each of the RNAi components during sexual development. Surprisingly, we observed a modest increase in the expression of the RNAi components during sexual development compared to vegetative growth (Fig 5C). In previous studies, northern blot analysis was employed to investigate the expression of these genes during vegetative growth and mating, and the modest 2- to 4-fold increase we observed using RT-PCR was possibly below the level of detection by northern blot. However, deletion of *ZNF3* and *QIP1* did not alter the expression of *RDP1* or *AGO1*, suggesting that Znf3 and Qip1 do not act as transcriptional regulators of the canonical RNAi components or mediate the modest increase in expression we observed in mating conditions We also investigated the expression of *ZNF3* and *QIP1* in the absence of the canonical RNAi components during sexual development. Deletion of *RDP1* did not affect the *ZNF3* transcript levels during sexual development, indicating that *RDP1* does not control the expression of this gene (Fig 5C). The expression of Znf3 was modestly but significantly increased in the *ago1*Δ mutants, which is the catalytic subunit of the RISC complex. Interestingly, expression of *QIP1* during sexual development decreased to vegetative levels in the absence of *RDP1*.

To explore a possible role of Znf3 in the translational regulation of the RNAi components, we deleted *ZNF3* and investigated the protein levels of Ago1 and Rdp1 fused with mCherry under the control of the endogenous promoter during sexual development. We detected a strong protein signal for both Ago1-mCherry and Rdp1-mCherry during sexual development with or without *ZNF3* (Fig 5D). Western blot analysis revealed that deletion of *ZNF3* resulted in a modest decrease in the protein abundance of Rdp1 under mating conditions (Fig 5E). It is possible that this decrease may not have been detectable via direct microscopy of cells expressing the Rdp1-mCherry fusion protein (Fig 5D). These results indicate that although Znf3, is not involved in transcriptional regulation of the canonical RNAi components, it could be involved in either translational regulation or in modulating protein stability via a role as a scaffolding protein.

### Znf3 localizes in P-bodies while Qip1 migrates to the nucleus during vegetative growth

RNAi silencing is a multifunctional pathway and different steps occur at different sites within the cell. The presence of tandem repeated genes or retrotransposons in the genome induces the transcription of aberrant ssRNA in the nucleus through an unknown mechanism and Rdp1 generates dsRNA from these sequences and evokes the RNAi pathway. The dsRNA travels to P-bodies, where processing and RNA silencing occurs. Dcr1/2 and Ago1, which localize to P-bodies, generate siRNAs that target mRNAs with complementary sequences for degradation [10]. These findings suggest that additional components of the pathway will localize either to the nucleus or to P-bodies.

**Fig 6:**
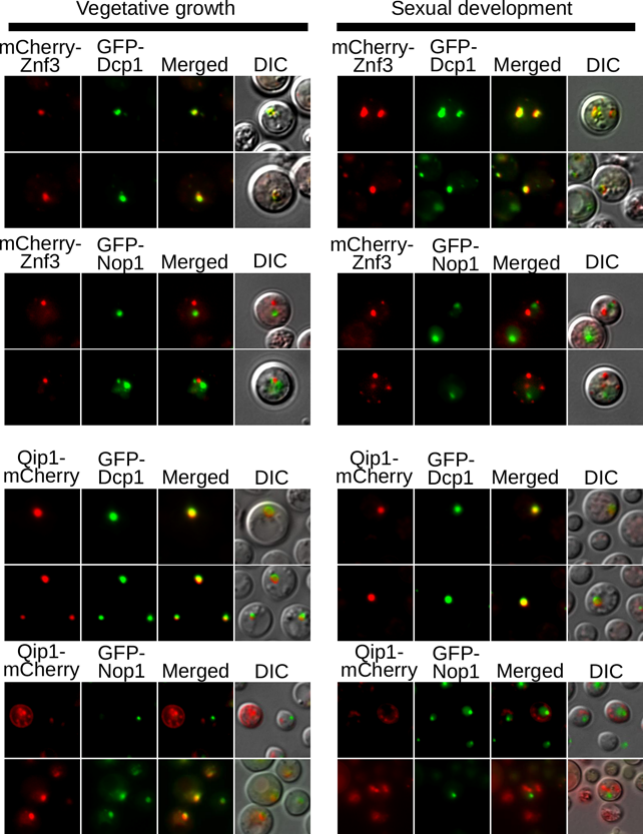
Znf3 and Qip1 localize to P-bodies during mating. Direct fluorescence microscopy of mCherry-Znf3 (A) or Qip1-mCherry (B) in strains with either GFP-tagged Nop1 or GFP-tagged Dcp1 was carried out in both vegetative growth and mating conditions. Znf3 localizes exclusively to P-bodies, marked by Dcp1, during mating. Qip1 localizes to either the nucleus or P-bodies during vegetative growth, but moves to P-bodies exclusively during mating. Strains used (A): MF156, MF162, and YL99**a** (B): MF228, MF237, and YL99**a**.

Znf3 has two NLS signals, therefore we initially hypothesized that Znf3 might localize to the nucleus where it could act as a transcription factor, or bind and degrade dsRNAs generated by Rdp1. To investigate the localization of Znf3, and because endogenous C-terminal tagging had failed to produce functional protein, the N-terminus of the protein was fused to mCherry, and expressed from the constitutively active *GPD1* promoter. The H99α and JF289**a** strains were transformed with the mCherry-Znf3 plasmid and evaluated by direct fluorescence microscopy. Surprisingly, we observed multiple bright foci in the cells indicating that the protein was present in more than one cellular compartment during sexual development (Fig 6A). To determine this cellular localization, we utilized two established marker components, one for P-bodies and the other for the nucleus. Dcp1, found in P-bodies, is responsible for decapping mRNAs during exonucleolytic degradation, while Nop1 is a component of the small subunit processome (a ribosome assembly intermediate) complex of the nucleolus [10,40]. GFP-Dcp1 and GFP-Nop1 were expressed from plasmids that were ectopically introduced into the genomes of strains expressing the mCherry-Znf3 protein and localization was observed during vegetative growth and sexual development. Surprisingly, we found that Znf3 localizes only in the P-bodies during both vegetative growth and sexual reproduction, despite the putative NLS signals (Fig 6A). These results suggest that Znf3 may participate directly in the RNAi silencing process and it may represent a novel element of the RNAi pathway.

Previous studies found that Qip1 localizes in the nucleus and that it physically interacts with Rdp1 and Ago1 during vegetative growth [30]. Although Ago1 is primarily localized in P-bodies during mating, where RNA silencing occurs, it has been also reported in the nucleus under vegetative growth conditions [30]. To further investigate the localization of Qip1, the protein was fused at the C-terminus with mCherry and expressed from the endogenous *QIP1* promoter. The fluorescent signal was evaluated via microscopy during vegetative growth and sexual development. Co-localization of Qip1-mCherry with GFP-Dcp1 or GFP-Nop1 revealed surprising results. During sexual development, where the RNAi pathway is highly induced, Qip1 was localized exclusively in P-bodies, potentially reflecting a role in RNA degradation (Fig 6B). During vegetative growth we observed Qip1 in association with either the P-bodies or the nucleus (Fig 6B). These results suggest that Qip1 may interact with Rdp1 in the nucleus during vegetative growth, possibly as a component of the SCANR complex to participate in the processing of the stalled splicing intermediate [30]. During mating Qip1 migrates to the P-bodies where it may subserve its conserved role in the RISC complex.

### Cpr2 contributes to the induction of Sex-induced Silencing through the same pathway as Ste3

**Fig 7:**
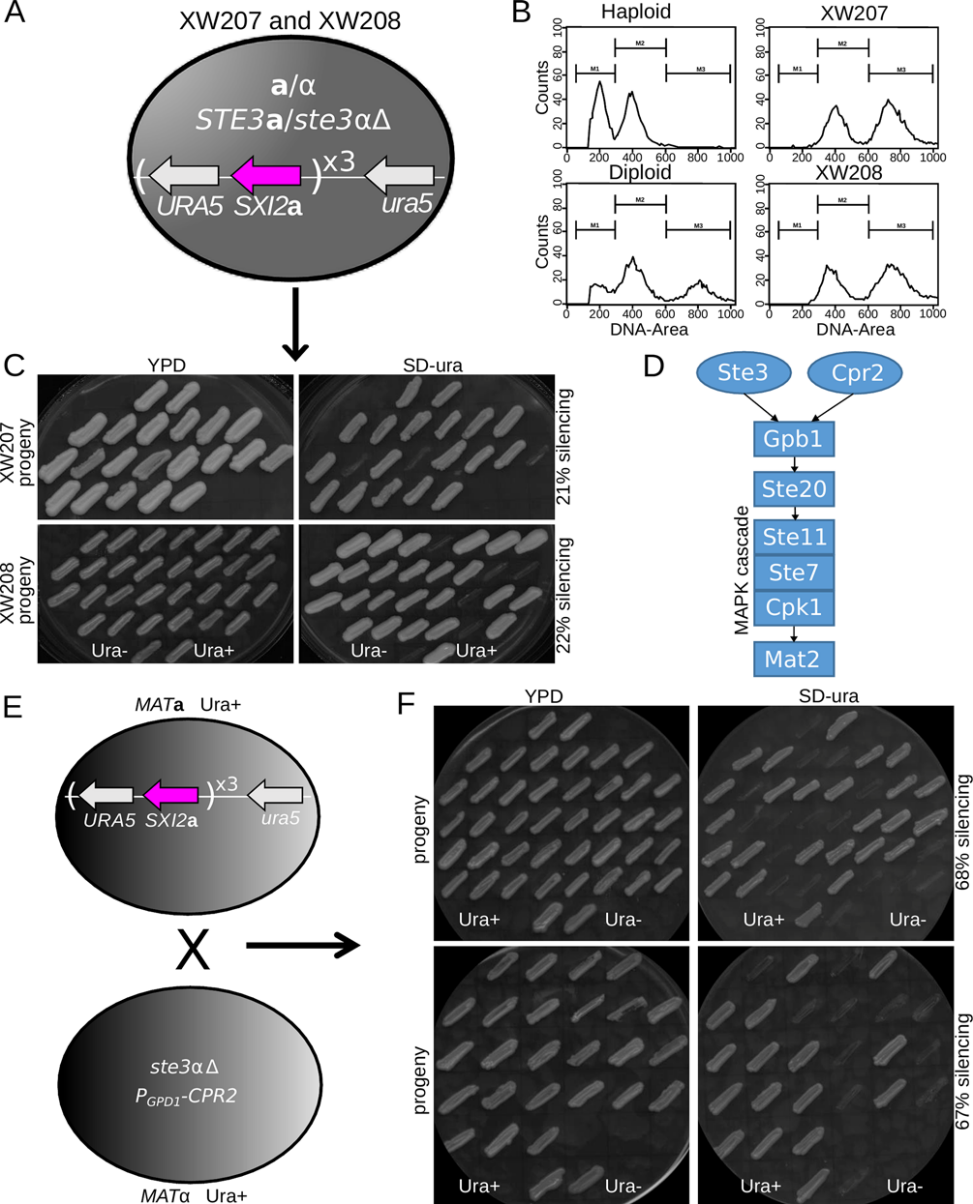
Cpr2 and Ste3 play redundant roles in intiating RNAi during SIS. (A) Schematic of the genotype of a diploid in which the *STE3*α gene was deleted. (B) Two independent *ste3*α/*STE3***a** mutants remain diploid based on FACS analysis of propidium iodide stained cells. Strains used: XW207 and XW208. (C) When sporulated, the progeny from both *ste3*αΔ/*STE3***a** mutant strains XW207 and XW208 show defects in SIS, with only 21% and 22% silencing, respectively, compared to ~50% in the wild type. Strains used: XW207 and XW208. (D) Schematic of the MAPK cascade triggered by Ste3 and Cpr2. (E) The *MAT***a** JF289 strain was crossed with a *MAT*α *ste3*αΔ strain that contained the *CPR2* gene under the control of the constitutively active *GPD1* promoter. (F) Analysis of progeny from the cross in (E) showed overexpression of *CPR2* suppresses the SIS silencing defect of the *ste3*α mutant. Strains used: XW209 and XW210.

We observed that both *cpr2*Δ and *fzc28*Δ mutants had defects in SIS but not in MIS. This suggests that these two pathways may differ in more than just their efficiency. As a result, we sought to test whether the role of Cpr2 in SIS was linked to its role in mating or independent of this function. We tested this by analyzing mutants lacking Ste3α, a pheromone receptor that shares the same G proteins as Cpr2. Ste3α mutants fail to mate, so a deletion was instead constructed in an **a**/α diploid (Fig 7A). FACS was used to verify that two independent *ste3*αΔ/**a** deletions remained stably diploid (Fig 7B). The *ste3*αΔ/**a** diploids were then sporulated and the progeny were dissected and tested for silencing of the *URA5* transgene. Both independent mutants demonstrated a defect in SIS, with a silencing frequency of only ~21–22% compared to 50% silencing in WT crosses.

To test whether this effect was mediated by the shared downstream MAP kinase cascade (Fig 7D), we utilized a *ste3*αΔ mutant complemented with an overexpressed *CPR2* gene under the control of the *GPD1* promoter to test whether ectopic overexpression of Cpr2 could compensate for the *ste3*αΔ mutant defect in SIS. This strain was mated with the JF289**a** *SXI2***a***-URA5* transgene array strain and spores were dissected, germinated, and phenotyped (Fig 7E and 7F). Overexpression of Cpr2 restored the SIS efficiency of the *ste3*αΔ/**a** mutant to ~ 67–68%. This suggests that both Cpr2 and Ste3 act coordinately to induce the RNAi pathway during the sexual cycle, and may not act as essential RNAi components themselves. Similarly, the transcription factor Fzc28 is a candidate to be the downstream effector of the Ste3/Cpr2 pathway, as it has no MIS defect but an absolute defect in SIS, suggesting it is essential for that arm of the pathway.

## Discussion

In this study we found that four novel proteins are required for silencing of the *SXI2***a**-*URA5* transgene during sexual development (SIS) and/or vegetative growth (MIS), and that they fall into two distinct classes: proteins essential for both RNAi pathways, and proteins influencing just the sex-induced arm of the pathway. Deletion analysis reveals that Znf3 and Qip1 are required for MIS and SIS RNAi silencing, similar to the canonical RNAi component Rdp1, while Cpr2 and the novel transcription factor Fzc28 influence only in SIS.

In further support of this model we found that *rdp1*Δ and *znf3*Δ mutants have very similar transcript profiles during mating characterized by increased abundance of messages from retrotransposons and other transposon-related genes. However, unlike RNAi components, whose expression remains largely stable during mitotic growth and mating, *ZNF3* and *QIP1* are transcriptionally induced during the sexual cycle. Although Znf3 has two NLS tags, we found that it localizes in P-bodies, where Dcr1/2 and Ago1 are also localized. Qip1 also localizes in P-bodies during sexual development; however, in some cases it migrates to the nucleus during vegetative growth. This may indicate that these two proteins act mechanistically in the silencing pathway, rather than as regulators of the canonical components.

RNA silencing is a highly conserved mechanism of transcriptional regulation. Since its discovery in *C*. *elegans* it has been identified in numerous species throughout the eukaryotic kingdom and it is hypothesized to be an ancestral feature of the last common eukaryotic ancestor [13,14,41]. An RNAi-related phenomenon was initially identified in plants and fungi, and later multiple species have been found to undergo RNA silencing mechanisms, including the fungi *Neurospora crassa*, *Mucor circinelloides*, and *Schizosaccharomyzes pombe* [20,42–44]. However, RNAi has been independently lost in some species, such as *Saccharomyces cerevisiae* and *Ustilago maydis*, which are missing all of the components of the RNAi pathway [18,25]. Nevertheless, the closely related species of *S*. *castellii* and *C*. *albicans* retain some of the RNAi components and substitute for the absence of others by employing noncanonical factors to produce dsRNA and shRNAs that map to transposable elements [45]. As a result, it is possible to learn more about the intact RNAi pathway of a species by comparing it to a related species that has lost some or all of its RNAi components [16,26]. This study shows that examining individual cases of RNAi loss with available sequenced genomes for closely related organisms is likely to be fruitful. The *Ustilago* clade is another basidiomycete example to which this approach could be applied [27].

In this study we identified a number of novel RNAi components, three of which, Cpr2, Znf3, and Fzc28, had no functional domains or similarity to a known RNAi component that would have suggested they might be involved in an RNAi pathway. Notably, there are two known canonical RNAi components that we did not identify using this approach: Ago2 and Dcr2. In the first case, this is because Ago2 is not conserved across all of the *Cryptococcus* RNAi-proficient genomes, as it is missing from the H99 *Cryptococcus neoformans* reference genome. In the second case, Dcr2 is retained in *C*. *deuterogattii*, which may suggest either that Dcr2 already has an additional non-RNAi role in *Cryptococcus*, that it has acquired a second role during the loss of RNAi and speciation of *C*. *deuterogattii*, or that the entire RNAi pathway has not been lost. As a precedent for the first two hypotheses, in *C*. *albicans*, a noncanonical Dicer plays a role in snRNA processing [46].

Also notable is that our screen identified components that play a role in only the sex-induced pathway but not in mitotic silencing. Loss of these components suggests one of two hypotheses regarding the RNAi loss: either loss of RNAi began with the inactivation of SIS, and without SIS the evolutionary pressure to maintain the MIS pathway was no longer strong enough to prevent loss of core components, or alternatively, loss began with the core mechanistic components involved in the vegetative arm, and the pressure to maintain the specialized regulatory machinery of SIS vanished, allowing loss of both *CPR2* and *FCZ28*. The latter case seems potentially more likely, as the loss of canonical RNAi components can allow relatively robust transposon movement even without undergoing the sexual cycle [30,47]. In addition, the loss of these SIS-specific components also provides an opportunity to elucidate the signaling processes connecting mating to induction of the RNAi pathway.

It is also interesting that the *Cryptococcus* species that has lost RNAi, *C*. *deuterogattii*, is the species causing an ongoing outbreak in the Pacific Northwest. The loss of RNA silencing is possibly associated with higher virulence in this strain, but because *C*. *neoformans* or *C*. *deneoformans* strains missing RNAi elements are not altered in virulence in a murine host [10], the loss of RNAi in the *C*. *deuterogattii* lineage may have instead had a longer-term impact on virulence trajectory. Indeed, loss of RNAi liberates transposons in *Cryptococcus* [10,30,47], which could provide adaptive benefits through the generation of increased genetic and phenotypic diversity.

We showed herein that Znf3 and Qip1 influence transgene induced silencing during mitotic growth and sexual reproduction. Deletion of these genes severely impaired silencing efficiency, even in unilateral crosses where only one parent was mutant. This phenotype is similar to *rdp1*Δ mutations, which abolish silencing during unisexual mating and impact silencing efficiency in unilateral bisexual crosses [10]. Given that Rdp1 is a major component of RNAi silencing, and that it is responsible for the initial steps generating dsRNAs, we propose that Znf3 also plays an important role in the pathway. Although the RNAi components Dcr1/2 and Ago1 are required for SIS, their deletion in a unilateral cross only lowers the silencing efficiency, indicating that their role may be redundant or largely complemented when one wild type nucleus is still present. Therefore, it is possible that Znf3 interacts with these proteins and may participate in the formation of the RISC complex. Znf3 is a large protein (~1515 aa), and so it could act as a scaffold to bring components of the RNAi pathway together in complex with the dsRNA substrate. Localization of Znf3 in P-bodies, where Dcr1/2 and Ago1 process the dsRNAs, may further support this hypothesis. Further, we provided evidence that Znf3 may play a role in protein translation or protein stability, which could be through a role as a scaffold in the P-bodies, or even as an RNA-binding protein through its zinc finger motifs.

Dumesic *et al*. showed that Qip1 localizes in both the cytoplasm and the nucleus during growth in rich media, and that it physically interacts with both Rdp1 and Ago1 [30]. We further confirmed that Qip1 localizes in the nucleus during vegetative growth; however, we showed that during sexual development Qip1 expression is highly induced and it is localized in P-bodies during sexual development. Possibly, Qip1 enhances the function of Rdp1 in the nucleus during vegetative growth, by participating in the generation of aberrant dsRNAs from repetitive loci. During sexual development, where transposon movement is highly induced, Qip1 may resume its conserved role in RNA processing in P-bodies, where it may interact with Ago1 and participate in the cleavage of the passenger strand. Interestingly, Qip1 appears to play a role in meiosis, as bilateral mutant crosses failed to yield recombinant progeny. Lee *et al*. showed that Qip is also essential for meiotic silencing and meiosis in *N*. *crassa* [48]. However, the two proteins are significantly different and *C*. *neoformans* Qip1 does not contain a canonical exonuclease domain, unlike Qip. This may suggest that either Qip1 plays a different mechanistic role in *C*. *neoformans* than Qip in *N*. *crassa*, despite a similar phenotypic outcome, or that Qip1 may contain an unrecognized exonuclease domain.

Sex-induced silencing is an efficient mechanism that protects the genome against mobile elements. Previous studies showed that ~5% of the *Cryptococcus* genome consists of transposons that cluster together in blocks and reside in both telomeric and centromeric regions on the chromosomes [36]. Transposon activation and movement may drive genome instability and phenotypic variation. Wang *et al*. found that transposons are transcriptionally induced specifically during sexual development, but they are silenced post-transcriptionally by the RNAi pathway [10]. These results suggest that transposons are derepressed during mating, which could increase the mutational burden of the progeny unless counteracted by the SIS RNAi pathway. During unisexual reproduction this mechanism could generate *de novo* genotypic and phenotypic variation in clonal populations and enable rapid adaptation to new environments. Thus, loss of the RNAi components may confer a beneficial advantage in clonal mitotic or sexual populations.

## Material and Methods

### Strains and media

The strains and plasmids used in this study are listed in S1 Table. The strains were maintained in glycerol stocks at -80°C and grown on rich YPD media at 30°C (Yeast extract Peptone Dextrose). Strains with selectable markers were grown on YPD containing nourseothricin (NAT) and/or G418 (NEO). Uracil auxotrophic isolates were tested on both SD medium lacking uracil and synthetic medium containing 5-FOA (1 g/l). Mating assays were performed on 5% V8 juice agar medium (pH = 5 for *C*. *neoformans* or pH = 7 for C. *deneoformans*) or on MS (Murashige and Skoog) medium minus sucrose (Sigma-Aldrich). The mating cultures were incubated in the dark at room temperature for 1 week.

### Genetic crosses and spore dissection

To visualize and isolate spores, strains of interest were co-cultured on solid V8 medium for 2 weeks at room temperature in the dark without parafilm. Basidiospores from the edges of the colonies were randomly isolated using a microdissection microscope equipped with a 25-μm microneedle (Cora Styles Needles ‘N Blocks, Dissection Needle Kit) as previously described [49]. Following germination the colonies were tested on YPD, YPD + NAT or NEO, SD-ura, and 5-FOA media. Genomic DNA was isolated using a CTAB protocol as previously described [50]. The presence of the *SXI2***a**-*URA5* transgene in the progeny was assessed by PCR using the primer pair JOHE16835/JOHE16836. Example gel images can be found in S3 Fig for the unilateral crosses depicted in Fig 2.

### Gene disruption

The gene of interest was disrupted using a standard overlap PCR approach described previously [17]. Briefly, the 5’ and 3’ flanking regions of the *ZNF3*, *QIP1*, and *CPR2* genes were amplified from H99α genomic DNA, and the selectable markers *NAT* and *NEO* were amplified from plasmids pAI3 and pJAF1, respectively. The flanking sequences and the selectable markers were used to generate a full-length deletion cassette in an overlap PCR reaction with the flanking primers. The deletion cassettes were introduced into the H99α and JF289**a** strains by biolistic transformation [51]. Gene replacement via homologous recombination was confirmed by PCR and Southern hybridization. The primers used to generate the deletion mutants are listed in S2 Table. All deletions were constructed from at least two independent cultures, inoculated from different single isolated colonies of the parent strain, and independent mutants were isolated from different transformations.

For the *fzc47*Δ and *fzc*28Δ mutants, two independent deletions were available in the KN99 background from a recent deletion collection [31]. They were crossed with JF289**a** and spores were dissected to isolate segregants that inherited the deletion, the transgene array, and were *MAT***a**. These segregants were named strains SEC5, SEC6, SEC7, and SEC8.

### Construction of strains for protein localization

To determine the cellular localization of the Znf3 protein, the mCherry protein was fused to the C-terminus of the protein under the control of the endogenous promoter using a standard overlap PCR approach. Briefly, 1 kb of sequence upstream of the start codon and 1 kb downstream of the stop codon were amplified from the wild type strain H99 genomic DNA using primers listed in S2 Table. The mCherry sequence fused with the NEO marker was amplified from plasmid pLKB25. The flanking sequences and the fluorescence marker were combined as a template for an overlap PCR reaction. The overlap PCR products were introduced into the H99α and JF289a strains by biolistic transformation. Transformants were analyzed by PCR and Southern hybridization.

To construct a plasmid encoding mCherry-Znf3, the mCherry protein was fused to the N-terminus of Znf3 under the control of the constitutively active *GPD1* promoter. The genomic sequence of *ZNF3* was amplified from H99α genomic DNA using primer pair JOHE37890/JOHE37891 and cloned into plasmid pLKB49 [52] digested with XbaI and PacI. Plasmid pMF81 was introduced into the H99α and JF289**a** strains by biolistic transformation and the transformants were screened by PCR and direct fluorescence microscopy. Stable transformants expressing *mCherry*-*ZNF3* and *QIP1-mCherry* were also transformed via ectopic insertion with the pXW11 (*GFP-DCP1*) and pSL04 (*GFP-NOP1*) plasmids to visualize P-bodies and the nucleus, respectively.

### Microscopy

To visualize mCherry-Znf3 and Qip1-mCherry together with GFP-Dcp1 and GFP-Nop1, the strains of interest were grown on YPD medium to determine localization during vegetative growth or mixed with the opposite mating-type strain on V8 medium for 24 hours to visualize the proteins during sexual reproduction. Briefly, cells were grown overnight in liquid YPD and washed with PBS. Cells were then counted and mixed in equal proportions of *MAT***a** and *MAT*α and spotted on V8 pH5 medium. These plates were incubated in the dark at room temperature for 24 hours. Cells were then scraped from the plates and mixed into sterile water and placed on prepared slides covered with 1.5% water agar. Imaging was performed with a Zeiss Axio Imager widefield fluorescence microscope at the Light Microscopy Core Facility at Duke University. Analysis was performed using the Metamorph Premier software package.

### Microarray and data analysis

For vegetative growth the cells were grown in 5 ml liquid YPD overnight at 30°C. The following day the cells were harvested, washed, frozen in liquid nitrogen, and lyophilized. Samples were kept at -80°C until analysis. To isolate RNA from mating assays, the desired α and **a** strains were grown in YPD liquid, washed with sterile water, mixed in equal amounts in eppendorf tubes, and a 1 ml suspension was spotted on V8 agar pH = 5 and incubated in the dark at room temperature for 24 hrs. The next day the mating cultures were harvested, washed with sterile water, frozen, lyophilized, and stored at -80°C. Total RNA was extracted using the RiboPure-Yeast Kit (Ambion) following the manufacturer’s instructions (Life Technologies #AM1926). Denaturing agarose gel electrophoresis and NanoDrop were used to assess quality and concentration of the RNA samples. The RNA was amplified using the Ambion® MessageAmp™ Premier RNA Amplification kit following the manufacturer’s instructions. cDNA was synthesized using AffinityScript reverse transcriptase (Stratagene), Cy3/Cy5 labeled, and hybridized to a *C*. *neoformans* microarray slide (*Cryptococcus* Community Microarray Consortium, Washington University, St. Louis, MO). Labeling and hybridization were conducted in the DNA Microarray Core Facility at Duke University. The slides were washed, scanned with a GenePix 4000B scanner (Axon Instruments), and processed with GenePix Pro (version 4.0). All microarrays were conducted in triplicate. GeneSpring software was used for statistical analysis employing Lowess normalization, reliable gene filtering, and ANOVA analysis (significance p<0.05).

### Comparative genomics screen

Published reference genomes from H99, B3501A, JEC21, and WM276 were compared with R265 using FungiDB (http://fungidb.org/fungidb/) [11,36,37,53]. Genes were selected that were present in all four of the non-*C*. *deuterogattii* reference genomes but absent or greater than 50% different in length in the R265 genome. Positive hits were manually examined using synteny maps produced by FungiDB in order to confirm that orthologs had been correctly identified. The majority of the initial hits were false positives attributable to sequencing gaps or incorrect gene annotation. For the remaining hits, sequences from all five reference genomes were manually aligned using Clustal [54] to verify that sequence deletions had occurred. In addition, *de novo* assemblies of 53 VGII genomes from previously published data were used to validate that deletions of all of the components were conserved in the VGII lineage and not restricted to the R265 genome [32,55].

### Calculation of Tajima’s D

Estimation of Tajima’s D was performed using a custom Perl script and the Bio::PopGen:Statistics package of BioPerl [56]. Briefly, genomes were aligned and SNPs were called as previously described [32]. To avoid sampling bias, a representative genome was chosen from each clonal expansion in the sequencing dataset for a total of 17 individual lineages out of the original 53 genomes. SNPs were sampled over a range using VCFTools [57], alternate references were constructed using GATK [58], and regions were aligned using ClustalW [54]. These alignments were imported by Bio::AlignIO and sampled using Bio::PopGen:Statistics [59] within BioPerl. To sample the genome as a whole, 2 kb windows every 10 kb throughout the genome were chosen. Locations with missing data for an individual were discarded, resulting in a total of 1159 data points.

## Supporting Information

S1 Fig Tajima’s D is not significantly changed in deletion remnants as compared to the whole genome. The distribution of estimates of the Tajima’s D statistic for the deletion sites is slightly more positive on average than the distribution of estimates for the genome as a whole, however the distributions are not statistically different (p = 0.0901). This suggests that the gene losses are relatively ancestral and any signal of bottleneck or population sweep attached to them has decayed out of the population.
(TIF)

S2 Fig Additional conserved genes identified as lost in *C*. *deuterogattii*. Gene losses in genes either shown elsewhere to influence RNAi, not tested here, or not influencing RNAi are visualized using ACT. Blastn alignments were utilized to align the H99 and R265 genomes.
(TIF)

S3 Fig Presence of transgene array as determined by PCR. Test for the presence of the *SXI2***a**-*URA5* transgene array via PCR. (A) Transgene array PCRs for progeny from the wild type H99 x JF289**a** cross depicted in Fig 2. (B) Transgene array PCRs for progeny from the unilateral H99 x *znf3*Δ cross depicted in Fig 2. Strains used: H99 x JF289 and H99 x XW205.
(TIF)

S4 Fig Non-canonical components are required for sex-induced silencing. (A) Progeny from wild type, unilateral mutant matings (one parent is mutant), and bilateral matings (both parents mutant) were isolated and evaluated for *URA5* silencing by growth on rich media (YPD) and SD-uracil. The parental and *ura5* mutant strains were included as controls. The plates were incubated at 30°C for 3 days. This data is quantified in Fig 2C. Strains used in S4 Fig: for WT: H99 x JF289**a**, for *znf3*Δ: MF65 x XW205, for *cpr2*Δ: YPH16 x JF289**a** and YPH16 x XW197, for *fzc47*Δ: YSB1406 x JF289**a** and YSB1407 x SEC5, for *fzc28*Δ: YSB2337 x JF289**a** and YSB2338 x SEC7. (B) While a unilateral cross of the *qip1* mutant showed a silencing defect, a bilateral cross produced substantially fewer spore chains and basidia, and spores from the *qip1* x *qip1* crosses did not germinate. Strains used for *qip1*Δ: SEC1 x H99 and SEC1 x SEC4.
(TIF)

S5 Fig Transposon quantity is largely, although not entirely, diminished in *C*. *deuterogattii*. A blastn search was conducted using a *C*. *neoformans* TE library [39] against the H99, WM276, and R265 reference genomes. The majority of TE elements have reduced numbers of blast hits in R265 as compared to the other genomes, although TCN3 and TCN6 are better conserved in copy number and TCN4 and LTR13 appear to be increased in copy number relative to WM276.
(TIF)

S1 Table Strains and plasmids used in this study. (DOCX)

S2 Table Oligonucleotides used in this study. (DOCX)

S3 Table Transposons overexpressed in *rdp1*Δ and *znf3*Δ mutant crosses relative to wild type crosses. (DOCX)
