# Supplementary figures and images for: Gene Network Polymorphism Illuminates Loss and Retention of Novel RNAi Silencing Components in the Cryptococcus Pathogenic Species Complex
Source: PLoS Genet. 2016 Mar 4;12(3):e1005868. doi: 10.1371/journal.pgen.1005868 (PMC4778953; doi:10.1371/journal.pgen.1005868)

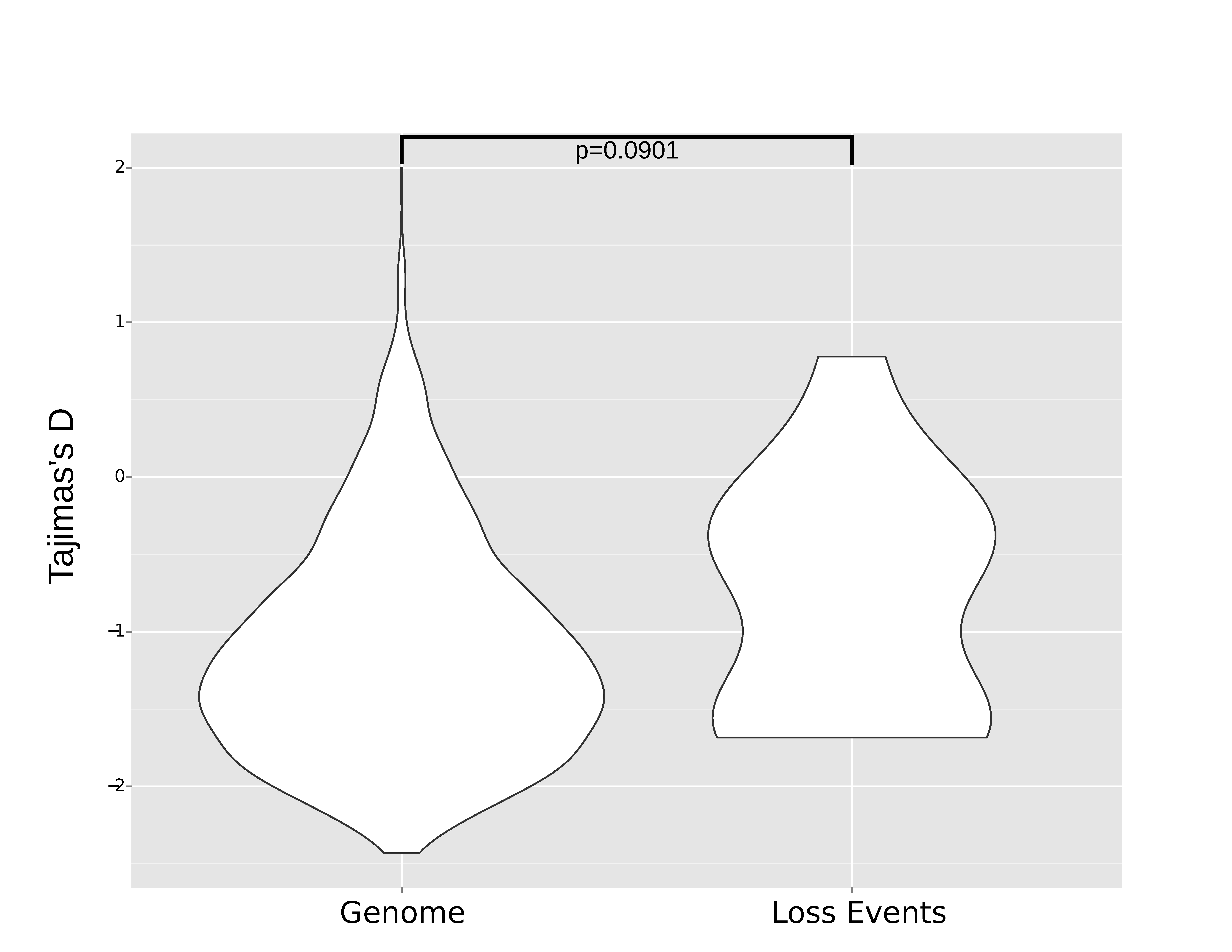

Supplement: S1 Fig — The distribution of estimates of the Tajima’s D statistic for the deletion sites is slightly more positive on average than the distribution of estimates for the genome as a whole, however the distributions are not statistically different (p = 0.0901). This suggests that the gene losses are relatively ancestral and any signal of bottleneck or population sweep attached to them has decayed out of the population. (TIF) [file pgen.1005868.s001.tif]

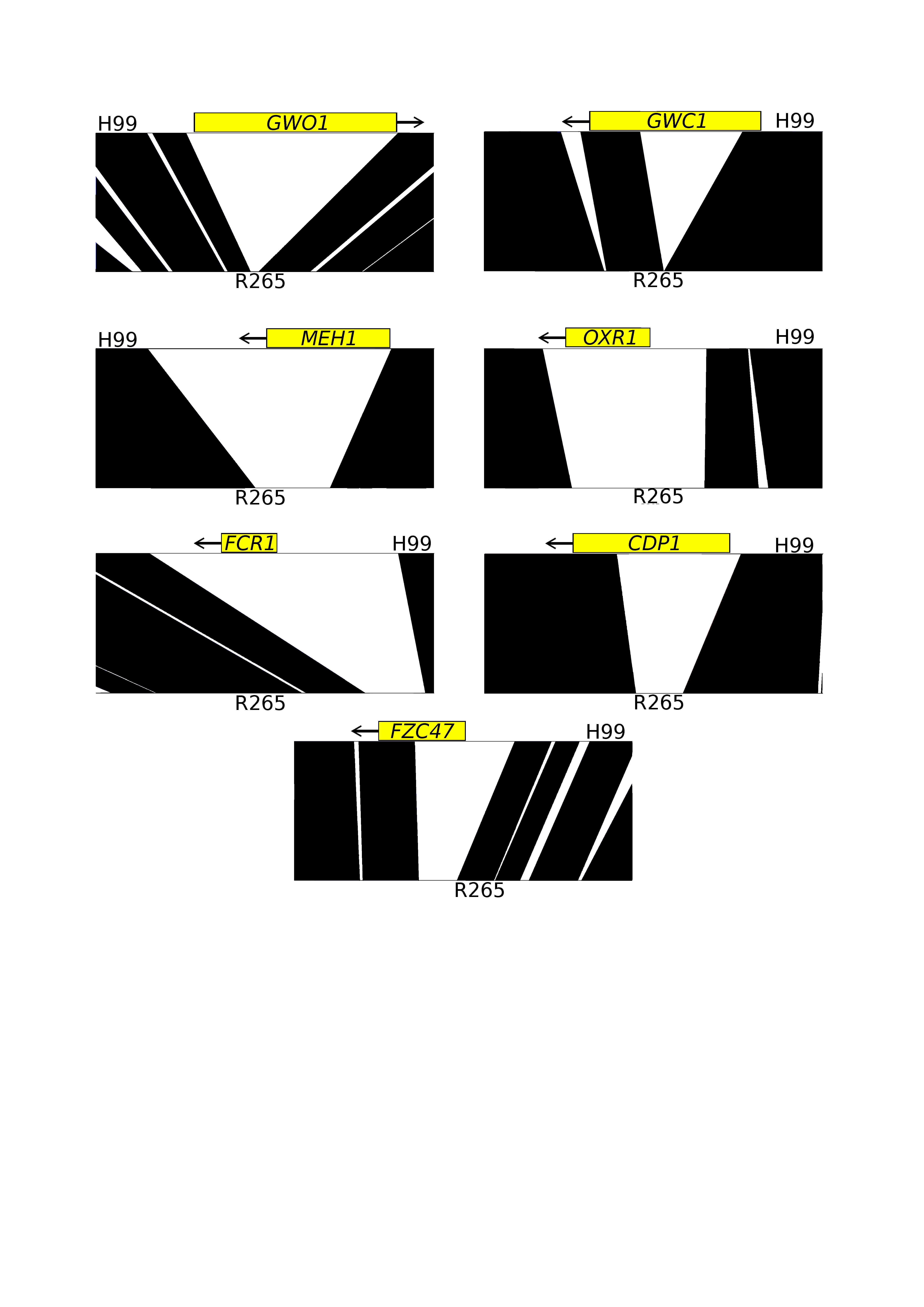

Supplement: S2 Fig — Gene losses in genes either shown elsewhere to influence RNAi, not tested here, or not influencing RNAi are visualized using ACT. Blastn alignments were utilized to align the H99 and R265 genomes. (TIF) [file pgen.1005868.s002.tif]

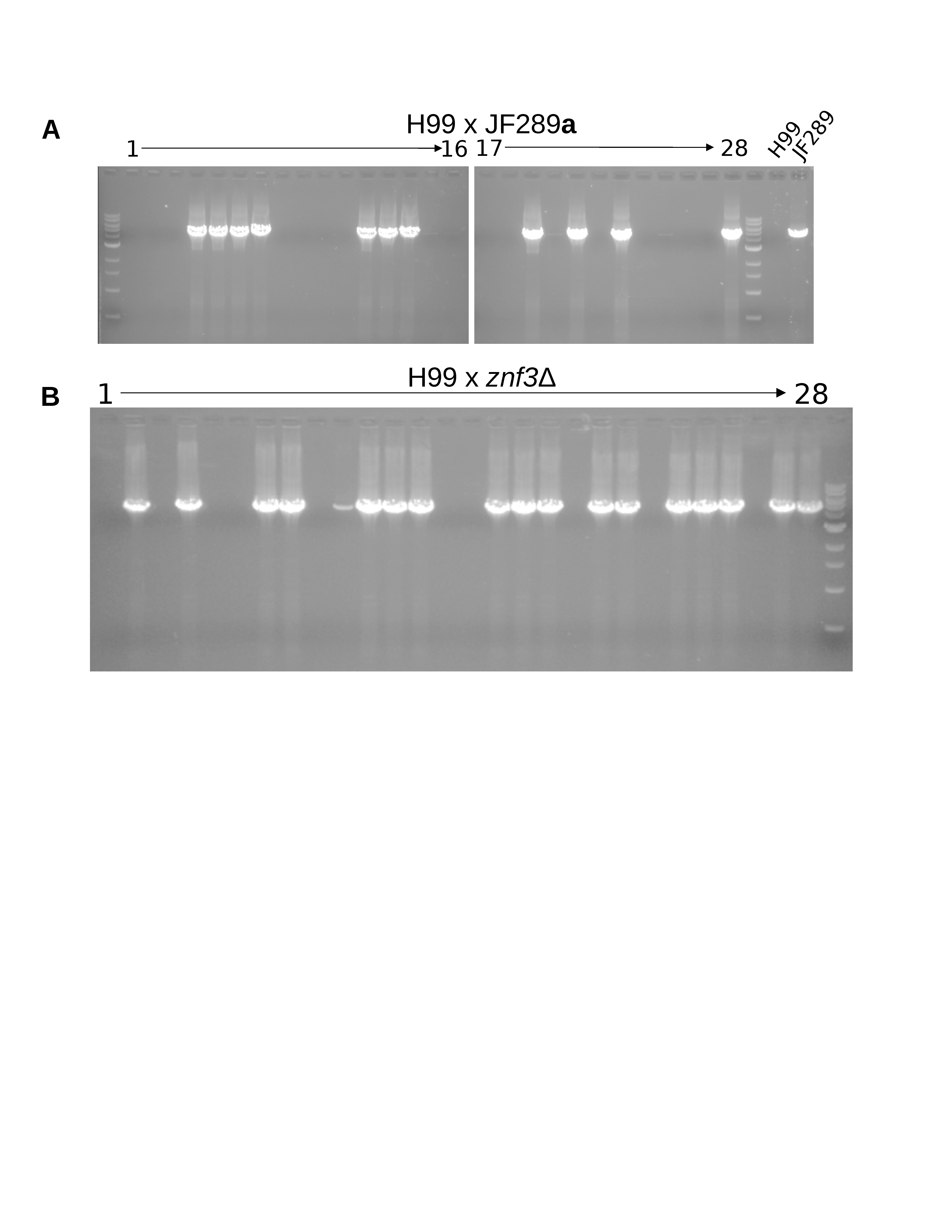

Supplement: S3 Fig — Test for the presence of the SXI2a-URA5 transgene array via PCR. (A) Transgene array PCRs for progeny from the wild type H99 x JF289a cross depicted in Fig 2. (B) Transgene array PCRs for progeny from the unilateral H99 x znf3Δ cross depicted in Fig 2. Strains used: H99 x JF289 and H99 x XW205. (TIF) [file pgen.1005868.s003.tif]

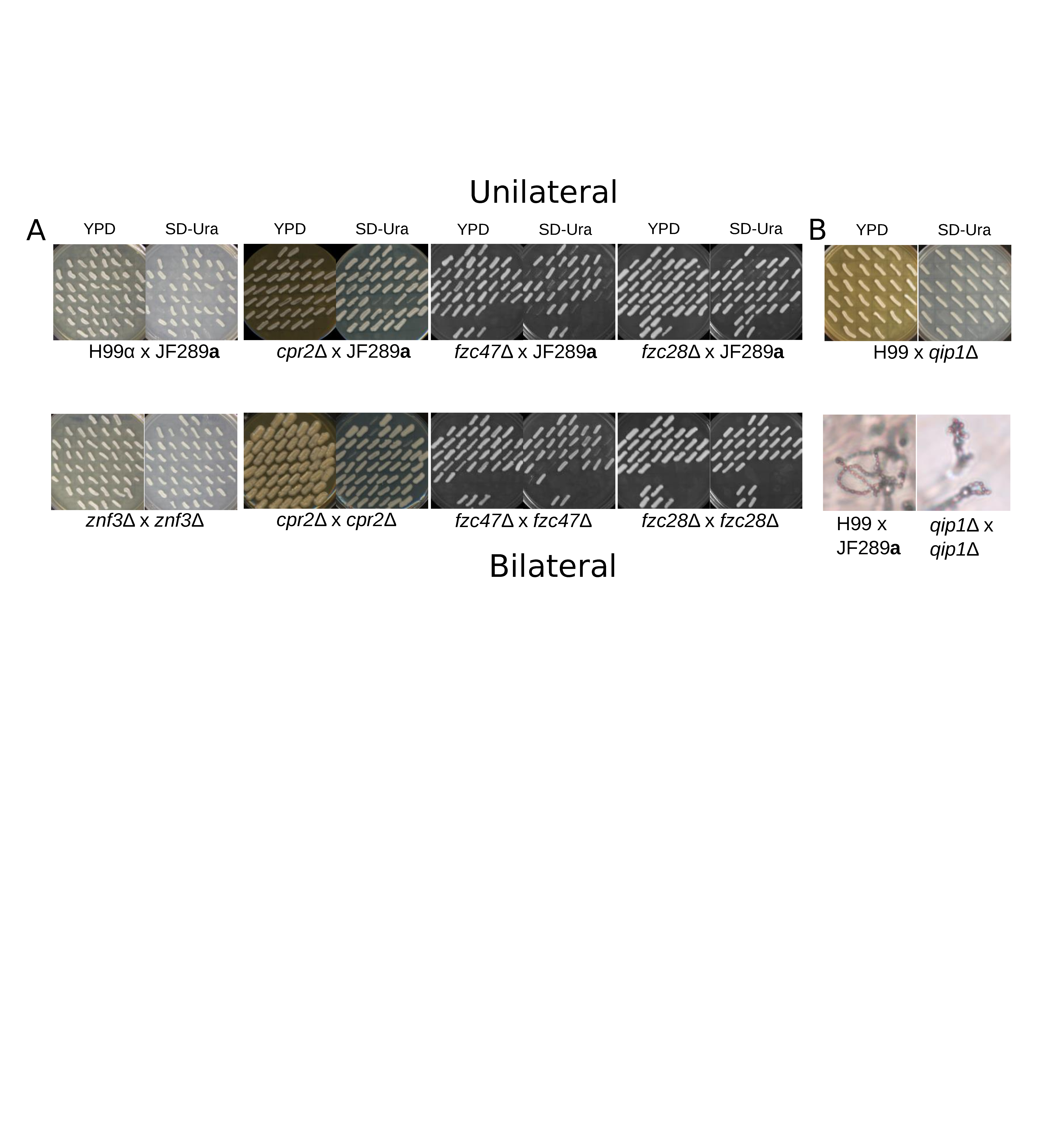

Supplement: S4 Fig — (A) Progeny from wild type, unilateral mutant matings (one parent is mutant), and bilateral matings (both parents mutant) were isolated and evaluated for URA5 silencing by growth on rich media (YPD) and SD-uracil. The parental and ura5 mutant strains were included as controls. The plates were incubated at 30°C for 3 days. This data is quantified in Fig 2C. Strains used in S4 Fig: for WT: H99 x JF289a, for znf3Δ: MF65 x XW205, for cpr2Δ: YPH16 x JF289a and YPH16 x XW197, for fzc47Δ: YSB1406 x JF289a and YSB1407 x SEC5, for fzc28Δ: YSB2337 x JF289a and YSB2338 x SEC7. (B) While a unilateral cross of the qip1 mutant showed a silencing defect, a bilateral cross produced substantially fewer spore chains and basidia, and spores from the qip1 x qip1 crosses did not germinate. Strains used for qip1Δ: SEC1 x H99 and SEC1 x SEC4. (TIF) [file pgen.1005868.s004.tif]

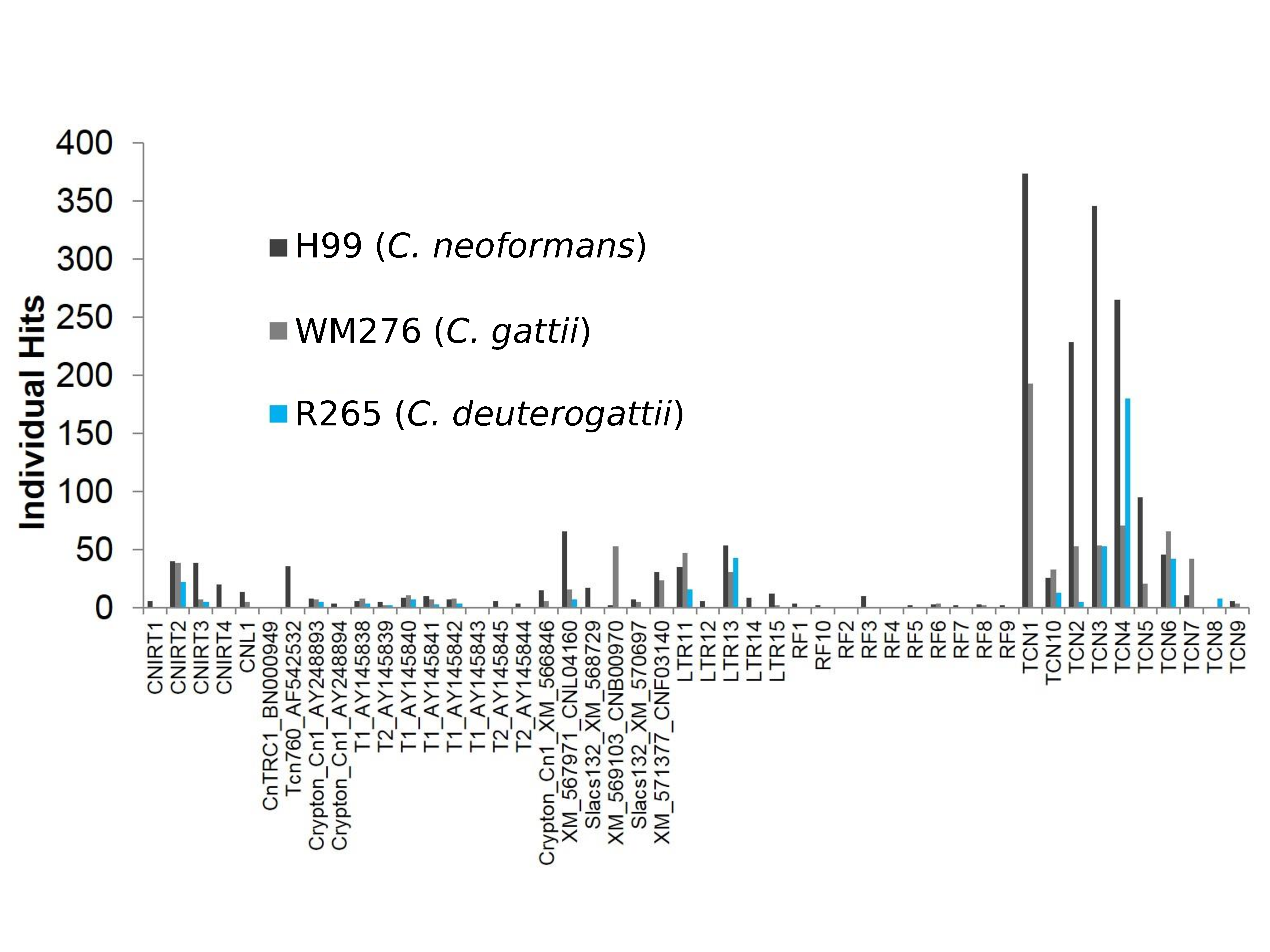

Supplement: S5 Fig — A blastn search was conducted using a C. neoformans TE library [39] against the H99, WM276, and R265 reference genomes. The majority of TE elements have reduced numbers of blast hits in R265 as compared to the other genomes, although TCN3 and TCN6 are better conserved in copy number and TCN4 and LTR13 appear to be increased in copy number relative to WM276. (TIF) [file pgen.1005868.s005.tif]
