# Supplementary material for: Gene Network Polymorphism Illuminates Loss and Retention of Novel RNAi Silencing Components in the Cryptococcus Pathogenic Species Complex
Source: PLoS Genet. 2016 Mar 4;12(3):e1005868. doi: 10.1371/journal.pgen.1005868 (PMC4778953; doi:10.1371/journal.pgen.1005868)
Supplement: S1 Table — (DOCX) [file pgen.1005868.s006.docx]

**S1 Table.** Strains and plasmids used in this study

| **Strain** | **Genotype** | **Source/Reference** |
| --- | --- | --- |
| *Cryptococcus neoformans* | | |
| H99 | *MAT*α | [46] |
| YL99**a** | *MAT***a** | [60] |
| JF289**a** | *MAT***a** *sxi2***a**Δ::*NAT* *ura5 SXI2***a**::*URA5*(x3) | [48] |
| YPH348 | *MAT***a** *rdp1*Δ::*NEO sxi2***a**Δ::*NAT* *ura5 SXI2***a**::*URA5*(x3) | [18] |
| YPH351 | *MAT*α *rdp1*Δ::*NEO* | [18] |
| YPH738 | *MAT***a** *ago1*Δ::*NEO sxi2***a**Δ::*NAT* *ura5 SXI2***a**::*URA5*(x3) | [18] |
| YSB299 | *MAT*α *ago1*Δ::*NEO* | [18] |
| YSB1406 | *MAT*α *fzc47*Δ::*NEO* | [31] |
| YSB1407 | *MAT*α *fzc47*Δ::*NEO* | [31] |
| YSB2337 | *MAT*α *fzc28*Δ::*NEO* | [31] |
| YSB2338 | *MAT*α *fzc28*Δ::*NEO* | [31] |
| YPH16 | *MAT*α *cpr2*Δ::*NAT* | [29] |
| XW197 | *MAT***a** *cpr2*Δ::*NAT sxi2***a**Δ::*NAT* *ura5 SXI2***a**::*URA5*(x3) | This study |
| XW198 | *MAT***a** *cpr2*Δ::*NAT sxi2***a**Δ::*NAT* *ura5 SXI2***a**::*URA5*(x3) | This study |
| XW207 | **a**/α diploid *ste3*αΔ *sxi2***a**Δ::*NAT* *ura5 SXI2***a**::*URA5*(x3) | This study |
| XW208 | **a**/α diploid *ste3*αΔ *sxi2***a**Δ::*NAT* *ura5 SXI2***a**::*URA5*(x3) | This study |
| XW209 | **a**/α diploid *ste3*αΔ P*_GPD1_*-*CPR2 sxi2***a**Δ::*NAT* *ura5 SXI2***a**::*URA5*(x3) | This study |
| XW210 | **a**/α diploid *ste3*αΔ P*_GPD1_*-*CPR2 sxi2***a**Δ::*NAT* *ura5 SXI2***a**::*URA5*(x3) | This study |
| SEC1 | *MAT***a** *qip1*Δ::*NEO sxi2***a**Δ::*NAT* *ura5 SXI2***a**::*URA5*(x3) | This study |
| SEC2 | *MAT***a** *qip1*Δ::*NEO sxi2***a**Δ::*NAT* *ura5 SXI2***a**::*URA5*(x3) | This study |
| SEC3 | *MAT*α *qip1*Δ::*NEO* | This study |
| SEC4 | *MAT*α *qip1*Δ::*NEO* | This study |
| SEC5 | *MAT***a** *fzc47*Δ::*NEO sxi2***a**Δ::*NAT* *ura5 SXI2***a**::*URA5*(x3) | This study |
| SEC6 | *MAT***a** *fzc47*Δ::*NEO sxi2***a**Δ::*NAT* *ura5 SXI2***a**::*URA5*(x3) | This study |
| SEC7 | *MAT***a** *fzc28*Δ::*NEO sxi2***a**Δ::*NAT* *ura5 SXI2***a**::*URA5*(x3) | This study |
| SEC8 | *MAT***a** *fzc28*Δ::*NEO sxi2***a**Δ::*NAT* *ura5 SXI2***a**::*URA5*(x3) | This study |
| MF62 | *MAT***a** *znf3*Δ::*NEO* | This study |
| XW205 | *MAT***a** *znf3*Δ::*NEO sxi2***a**Δ::*NAT* *ura5 SXI2***a**::*URA5*(x3) | This study |
| XW206 | *MAT***a** *znf3*Δ::*NEO sxi2***a**Δ::*NAT* *ura5 SXI2***a**::*URA5*(x3) | This study |
| MF65 | *MAT*α *znf3*Δ::*NEO* | This study |
| MF188 | *MAT***a** *sxi2***a**Δ::*NAT* *ura5 SXI2***a**::*URA5*(x3) *QIP1*::*mCherry*::*NEO* | This study |
| MF190 | *MAT*α *QIP1*::*mCherry*::*NEO* | This study |
| MF201 | *MAT*α *AGO1*::*mCherry*::*NEO znf3*Δ::*NAT* | This study |
| XW35 | *MAT*α *AGO1*::*mCherry*::*NEO* | [10] |
| XW37 | *MAT*α *RDP1*::*mCherry*::*NEO* | [10] |
| MF197 | *MAT*α *RDP1*::*mCherry*::*NEO znf3*Δ::*NAT* | This study |
| MF198 | *MAT*α *RDP1*::*mCherry*::*NEO znf3*Δ::*NAT* | This study |
| MF140 | *MAT*α P*_GPD1_*-*mCherry*::*ZNF3*::*NEO* | This study |
| MF153 | *MAT*α P*_GPD1_*-*mCherry*::*ZNF3*::*NEO* | This study |
| MF228 | *MAT*α *QIP1*::*mCherry*::*NEO GFP*::*DCP1*::*NAT* | This study |
| MF237 | *MAT*α *QIP1*::*mCherry*::*NEO GFP*::*NOP1*::*NAT* | This study |
| MF162 | *MAT*α P*_GPD1_*-*mCherry*::*ZNF3*::*NEO GFP*::*NOP1*::*NAT* | This study |
| MF156 | *MAT*α P*_GPD1_*-*mCherry*::*ZNF3*::*NEO GFP*::*DCP1*::*NAT* | This study |
| Plasmids |  |  |
| pXW11 | *GFP*::*DCP1*::*NAT Amp^R^* | [18] |
| pSL04 | *GFP*::*NOP1*::*NAT Amp^R^* | [40] |
| pLKB25 | P*_GPD1_*-*mCherry*::*NEO* | [61] |
| pLKB49 | P*_GPD1_*-*mCherry*::*NEO* | [52] |
| pMF81 | P*_GPD1_*-*mCherry*::*ZNF3*::*NEO* | This study |
|  |  |  |

**Supporting Information References**

60. Semighini CP, Averette AF, Perfect JR, Heitman J (2011) Deletion of *Cryptococcus neoformans* AIF ortholog promotes chromosome aneuploidy and fluconazole-resistance in a metacaspase-independent manner. PLoS Pathog 7: e1002364.

61. Kozubowski L, Heitman J (2010) Septins enforce morphogenetic events during sexual reproduction and contribute to virulence of *Cryptococcus neoformans*. Mol Microbiol 75: 658–675.
