# Supplementary material for: Gene Network Polymorphism Illuminates Loss and Retention of Novel RNAi Silencing Components in the Cryptococcus Pathogenic Species Complex
Source: PLoS Genet. 2016 Mar 4;12(3):e1005868. doi: 10.1371/journal.pgen.1005868 (PMC4778953; doi:10.1371/journal.pgen.1005868)
Supplement: S2 Table — (DOCX) [file pgen.1005868.s007.docx]

**S2 Table.** Oligonucleotides used in this study

| **Primer** | **Sequence (5’ to 3’)** | **Comment** |
| --- | --- | --- |
| M13F | GTAAAACGACGGCCAG | universal oligo |
| M13R | CAGGAAACAGCTATGAC | universal oligo |
| JOHE16835 | TTGGGTCAACTCTAATAAGGT | *SXI2***a**-*URA5* transgene screening [10] |
| JOHE16836 | TCTATGGCGAACGTGCTCATT | *SXI2***a**-*URA5* transgene screening [10] |
| JOHE21985 | TCTGAGTTTGGCGTGTCTGTCACT | *znf3* screening |
| JOHE21986 | TCTTCATAAGTGGCAGCGTGGACT | *znf3* screening |
| JOHE24058 | AAACAGAAGCAGCGTGATAAGGCGG | *ZNF3* deletion 5’ F |
| JOHE24059 | CTGGCCGTCGTTTTACTAGTAGCTGGCTGTTTCTAAAGATG | *ZNF3* deletion 5’ R |
| JOHE24060 | GTCATAGCTGTTTCCTGGGGGCAACAATCCATATAGCA | *ZNF3* deletion 3’ F |
| JOHE24061 | GAGAGAGTAGTAGTCGGGTTGGGTGGC | *ZNF3* deletion 3’ R |
| JOHE38286 | ACGACCAAGCATCCAACCTAG | *ZNF3-mCHERRY* C-terminus |
| JOHE38287 | CTCGCCCTTGCTCACCATCCAGCCACCAGTGTCTCC | *ZNF3-mCHERRY* C-terminus |
| JOHE38288 | GGAGACACTGGTGGCTGG ATGGTGAGCAAGGGCGAG | *ZNF3-mCHERRY* C-terminus |
| JOHE38318 | CTCCTCCTATCTTTTTTACCAAGCTTGGTACCGAGCTC | *ZNF3-mCHERRY* C-terminus |
| JOHE38319 | GAGCTCGGTACCAAGCTTGGTAAAAAAGATAGGAGGAG | *ZNF3-mCHERRY* C-terminus |
| JOHE21985 | TCTGAGTTTGGCGTGTCTGTCACT | *ZNF3-mCHERRY* screening |
| JOHE24061 | GAGAGAGTAGTAGTCGGGTTGGGTGGC | *ZNF3-mCHERRY* screening |
| JOHE38727 | GGAGTTCATGCGCTTCAAGGTG | *ZNF3-mCHERRY* RT-PCR |
| JOHE38728 | TTGTAGATGAACTCGCCGTCCTG | *ZNF3-mCHERRY* RT-PCR |
| JOHE39521 | CTCAACCTGTGGAAGCCACCA | *ZNF3-mCHERRY* sequencing |
| JOHE39522 | TGTTGGGAGGGAATCTGAACGAA | *ZNF3-mCHERRY* sequencing |
| JOHE39523 | TGTAGACGAGTCCGGTTGGAT | *ZNF3-mCHERRY* sequencing |
| JOHE39524 | GAGGTGATGTCCAACTTGATGTTGA | *ZNF3-mCHERRY* sequencing |
| JOHE39525 | ATCCCACGACGCGTTTTTGAC | *ZNF3-mCHERRY* sequencing |
| JOHE38790 | ATTGTATCTAGATATGCTCACAGCCTTCTTCGGCTA | *mCHERRY-QIP1* N-terminus |
| JOHE38791 | GCGTTAATTAAGAGAGAGTAGTAGTCGGGTTGGGT | *mCHERRY-QIP1* N-terminus |
| JOHE38792 | GGCTCTTCTTTGACAGGAGTTTCG | *ZNF3-mCHERRY* screening |
| JOHE38875 | GCATGTCTCATCTTCATCTTCTGG | *fzc47* screening |
| JOHE38876 | GTACTCCCAGTCACCTGCTCC | *fzc47* screening |
| JOHE38839 | GAGATCGTACGGCTCAGC | *fzc28* screening |
| JOHE38840 | GGCGAACATTCCTCTTGC | *fzc28* screening |
| JOHE39128 | GGCGATCGGCAAAGAGTTAC | *QIP1* deletion 5’ F |
| JOHE39129 | CTGGCCGTCGTTTTACGATCGGAATACAAAGAAGTCG | *QIP1* deletion 5’ R |
| JOHE39130 | GTCATAGCTGTTTCCTGATAATAACGACAAGTATCTTCATGAACAG | *QIP1* deletion 3’ F |
| JOHE39131 | CAGAGCAAAAAGTGGGAAGCG | *QIP1* deletion 3’ R |
| JOHE38869 | GGACGATAACAACCCAGGAGGC | *qip1* screening |
| JOHE38870 | CCTCTACGGCATCTAGAACGTTTG | *qip1* screening |
| JOHE40319 | CGAAGTGTGCAAGACCACATACTGG | *QIP1-mCHERRY* C-terminus |
| JOHE40320 | CTCGCCCTTGCTCACCATAACCTCTACGGCATCTAGAA | *QIP1-mCHERRY* C-terminus |
| JOHE40321 | TTCTAGATGCCGTAGAGGTTATGGTGAGCAAGGGCGAG | *QIP1-mCHERRY* C-terminus |
| JOHE40322 | CCTAGCTCCCTTCCTGGCCCAAGCTTGGTACCGAGCTC | *QIP1-mCHERRY* C-terminus |
| JOHE40323 | GAGCTCGGTACCAAGCTTGGGCCAGGAAGGGAGCTAGG | *QIP1-mCHERRY* C-terminus |
| JOHE40324 | GCTTCGATGTCAGAGTCAACAGAGC | *QIP1-mCHERRY* C-terminus |
| JOHE40325 | GCATCATTACCGGCATTACCGAC | *QIP1-mCHERRY* sequencing |
| JOHE40326 | GTAGAATCTTCCTACCTGCCGC | *QIP1-mCHERRY* sequencing |
| JOHE40327 | GTACATCCGCTCGGAGG | *QIP1-mCHERRY* sequencing |
| JOHE40328 | GGAGGGTGTACATCCTTTTCCG | *QIP1-mCHERRY* sequencing |
| JOHE40329 | TCTGAGCGTGACAAGGTGGG | *QIP1-mCHERRY* screening |
| JOHE40330 | TCCTCGAAGTTCATCACGCGC | *QIP1-mCHERRY* screening |
| JOHE40331 | CTGTGCTCGACGTTGTCACTG | *QIP1-mCHERRY* screening |
| JOHE40332 | CTTATCGCCGCAACGTGTCG | *QIP1-mCHERRY* screening |
| JOHE40347 | CGCTCCCGGTCTTTGACTCCA | *QIP1-mCHERRY* RT-PCR |
| JOHE40348 | TCCTCTCGTTCAACCCTTCGC | *QIP1-mCHERRY* RT-PCR |
