# Supplementary material for: Gene Network Polymorphism Illuminates Loss and Retention of Novel RNAi Silencing Components in the Cryptococcus Pathogenic Species Complex
Source: PLoS Genet. 2016 Mar 4;12(3):e1005868. doi: 10.1371/journal.pgen.1005868 (PMC4778953; doi:10.1371/journal.pgen.1005868)
Supplement: S3 Table — (DOCX) [file pgen.1005868.s008.docx]

**S3 Table.** Transposons overexpressed in *rdp1*Δ and *znf3*Δ mutant crosses relative to wild type crosses.

| **ID tag** | ***rdp1*Δ x *rdp1*Δ**  **a x α** | | ***znf3*Δ x *znf3*Δ**  **a x α** | | **Comment** |  |  |
| --- | --- | --- | --- | --- | --- | --- | --- |
| CNAG_01966  CNAG_06805  CNAG_05266  CNAG_02244  CNAG_00948  1772.seq.161  1621.seq.152  CNAG_01967  180.m00139  1662.seq.118  1761.seq.055  CNAG_04421  CNAG_04426  CNAG_00946  1743.seq.172  CNAG_00958  1751.seq.040  CNAG_07042  CNAG_00947  1672.seq.136  CNAG_06478  1621.seq.150  CNAG_06701  CNAG_00951  1641.seq.049  CNAG_02518  CNAG_02391  CNAG_02390  CNAG_02253  CNAG_06700  CNAG_02560  CNAG_02042  CNAG_06757  CNAG_06863  1742.seq.057  CNAG_01383  CNAG_05212  1702.seq.183 | \| 15.7 \| \| --- \| \| 15.59 \| \| 14.21 \| \| 13.96 \| \| 13.32 \| \| 12.19 \| \| 11.05 \| \| 10.31 \| \| 10.16 \| \| 9.321 \| \| 9.213 \| \| 8.795 \| \| 8.702 \| \| 7.735 \| \| 7.713 \| \| 7.663 \| \| 7.351 \| \| 7.31 \| \| 7.201 \| \| 7.106 \| \| 6.761 \| \| 6.524 \| \| 5.857 \| \| 5.713 \| \| 5.37 \| \| 5.29 \| \| 4.891 \| \| 4.851 \| \| 4.791 \| \| 4.477 \| \| 3.926 \| \| 3.673 \| \| 3.493 \| \| 3.419 \| \| 3.266 \| \| 3.256 \| \| 3.231 \| \| 3.134 \| | \| 11.44 \| \| --- \| \| 7.67 \| \| 9.174 \| \| 8.932 \| \| 10.7 \| \| 8.264 \| \| 6.577 \| \| 6.721 \| \| 6.888 \| \| 5.597 \| \| 5.415 \| \| 6.132 \| \| 7.056 \| \| 4.692 \| \| 5.209 \| \| 4.896 \| \| 6.056 \| \| 4.952 \| \| 3.313 \| \| 4.017 \| \| 5.612 \| \| 4.093 \| \| 3.679 \| \| 3.278 \| \| 5.077 \| \| 4.821 \| \| 4.073 \| \| 4.088 \| \| 3.477 \| \| 3.327 \| \| 3.693 \| \| 2.976 \| \| 3.068 \| \| 2.911 \| \| 1.859 \| \| 2.763 \| \| 2.664 \| \| 2.191 \| | | \| Tcn1 \| \| --- \| \| Hypothetical methyltransferase \| \| Putative acetate transporter \| \| Transposable element \| \| Transposable element \| \| Tcn1 \| \| Transposable element \| \| Transposable element \| \| Tcn2 \| \| Tcn1 \| \| Transposable element \| \| Transposable element \| \| Tcn1 \| \| Transposable element \| \| Tcn1 \| \| Tcn1 \| \| Tcn1 \| \| RNA helicase \| \| Tcn1 \| \| RNA-dependent DNA-polymerase \| \| Transposable element \| \| Tcn1 \| \| Transposable element \| \| Tcn3 \| \| Tcn1 \| \| Transposable element \| \| Transposable element \| \| Transposable element \| \| Tcn1 \| \| Transposable element \| \| Transposable element \| \| Transposable element \| \| RNA helicase \| \| Putative endonuclease \| \| RNA-dependent DNA-polymerase \| \| DNA helicase \| \| Retrotransposon nucleoside \| \| Tcn1 \| | | | |
|  |  |  | |  | | |  |
